# Effects of a future work self writing intervention on ruminative career exploration and career decision-making difficulties among final-year university students: a three-arm randomized controlled trial

**DOI:** 10.3389/fpsyg.2026.1965900

**Published:** 2026-10-07

**Authors:** Shang Sun, Di Yang

**Affiliations:** 1 School of Economics and Management, Anhui University of Science and Technology, Huainan, Anhui, China; 2 School of Electrical and Information Engineering, Anhui University of Science and Technology, Huainan, Anhui, China

**Keywords:** career decision-making difficulties, career exploration, career guidance, future work self salience, randomized controlled trial, ruminative career exploration, school-to-work transition, writing intervention

## Abstract

**Introduction:**

Career decision-making difficulties are widespread among final-year university students entering an uncertain labor market. A salient future work self is commonly assumed to relieve them by motivating career exploration, yet associations between exploration and indecision remain inconsistent across studies. Drawing on identity development theory, we distinguish reflective from ruminative career exploration, defined as repetitive self-questioning that generates distress rather than usable information, and test whether a future work self writing intervention works by decreasing rumination rather than by increasing exploration.

**Methods:**

In a three-arm parallel-group randomized controlled trial, 486 final-year students from 4 universities in China were allocated 1:1:1 to future work self writing, an active control (general future planning writing) or a waitlist control. The intervention comprised four weekly structured writing sessions of approximately 20 minutes, and outcomes were assessed at baseline, post-intervention, and one- and three-month follow-up. Primary outcomes were ruminative career exploration and the lack of readiness dimension of career decision-making difficulties; reflective exploration, the other two difficulty dimensions and future work self salience were secondary, and employment indicators exploratory. Intention-to-treat analyses used latent growth curve modeling and longitudinal mediation.

**Results:**

Relative to both control conditions, future work self writing reduced ruminative career exploration (*d* = 0.41, 95% CI [0.22, 0.61] vs. waitlist) and lack of readiness (*d* = 0.43, 95% CI [0.25, 0.62] vs. waitlist), estimated as slope contrasts across the four-wave observation period rather than as a test of the between-group difference at three months specifically. Effects on reflective career exploration were non-significant and statistically equivalent to zero within a margin of *d* = 0.30. Change in ruminative career exploration accounted for approximately 35% of the effect on lack of readiness, whereas change in reflective exploration did not; these estimates are associational rather than definitive evidence of causal mediation, and no employment indicator remained significant after correction.

**Discussion:**

The benefit of clarifying one's future work self appears to lie less in stimulating additional exploration than in interrupting unproductive career rumination. Career guidance may therefore be more effective when it addresses how students explore rather than how much.

## 1 Introduction

### 1.1 The school-to-work transition and career decision-making difficulties

The transition from higher education into work has become markedly less predictable over the past decade (Hirschi, 2018). Occupational structures shift faster than curricula adapt. Students are asked to commit to an occupational direction while the information relevant to that commitment keeps changing (Akkermans et al., 2020). Recent evidence confirms that labor market uncertainty continues to intensify career-related distress among university graduates in multiple national contexts (Xu, 2023a). Career decision-making difficulties are therefore common rather than exceptional among final-year students.

In the Chinese context these difficulties are particularly consequential. The number of university graduates in China reached 11.79 million in 2024 and has risen steadily in each preceding year (Wang and Wang, 2024). This growth has created sustained pressure on graduate labor markets and compelled the Ministry of Education to issue annual directives encouraging universities to strengthen career guidance services. These directives recommend the integration of career development courses as mandatory credit-bearing components of the curriculum.

Gati et al. (1996) organized career decision-making difficulties into a taxonomy with three superordinate categories. The first category is lack of readiness. It covers low motivation, general indecisiveness and dysfunctional beliefs about the decision process. The second category is lack of information. It covers insufficient knowledge about the decision process and about the self. It also covers insufficient knowledge about occupations and about ways of obtaining further information. The third category is inconsistent information. It covers unreliable information, internal conflicts and external conflicts. Saka et al. (2008) later showed that readiness-related difficulties behave differently from information-related difficulties. Readiness-related difficulties are relatively trait-like and resistant to informational input. Information-related difficulties respond more readily to targeted intervention. Recent work applying the dual-process theory of career decision-making has further demonstrated that these categories operate jointly through distinct processual mechanisms rather than independently (Xu, 2023b).

This distinction matters for intervention research. An intervention that supplies information should reduce the second category but leave the first category largely untouched. An intervention that changes how students process career-relevant thought should show a different pattern. Most published studies collapse the three categories into a single total score (Gati et al., 1996). That practice obscures precisely the evidence that would identify a mechanism. The present study therefore models the three categories separately.

Unresolved career indecision carries real costs. It predicts delayed labor market entry, poorer person-job fit after entry and elevated psychological distress during the transition (Guan et al., 2013). In the Chinese context these costs are concentrated within a compressed institutional window. Autumn recruitment runs from September through November. Spring recruitment runs from March through April. Graduation follows in June. This calendar creates a naturally structured developmental window that has no direct counterpart in most Western samples.

### 1.2 Future work self salience as a motivational resource

Strauss et al. (2012) introduced future work self salience to capture how clearly and how accessibly a person represents the self they hope to become at work. The construct has two components. Elaboration refers to how detailed and coherent the representation is. Accessibility refers to how easily the representation comes to mind. Future work self salience is distinct from career goals and from occupational aspirations. Goals specify outcomes whereas a future work self specifies an identity.

A recent systematic review by Talluri et al. (2025) synthesized the rapidly growing literature on future work self salience. The review identified proactive motivation theory (Parker et al., 2010) as the dominant theoretical framework. Within that framework a salient future work self supplies the reason to act. It converts a diffuse sense of the future into a standard against which present behavior can be evaluated. The discrepancy between the present self and the future work self is assumed to energize proactive career behavior. Taber and Blankemeyer (2015) reported that future work self salience predicted proactive career behaviors beyond career adaptability. Ling et al. (2022) demonstrated that future work self salience and future time perspective serially mediated the link between proactive personality and career adaptability among Chinese college students.

Several recent studies have extended these findings to new populations and outcomes. Zhang et al. (2022) found that future work self was linked to both career planning and career indecision among Chinese college students through the mediating role of career exploration. Voigt and Strauss (2024) showed that future work self salience moderated the effects of interacting with artificial intelligence on career-related cognition. These findings confirm the relevance of the construct but also highlight an unresolved question about the mechanism through which it affects decision-making.

Brief writing tasks have been used to make hoped-for identities more salient. The underlying paradigm derives from work on writing about life goals (King, 2001) and on visualizing a best possible self (Sheldon and Lyubomirsky, 2006). A recent systematic review of positive expressive writing interventions confirmed that best possible self writing produces the most consistent benefits for wellbeing and positive affect among the various writing paradigms (Hoult et al., 2025). These paradigms are inexpensive and easy to deliver at scale. Existing evaluations of future work self writing tasks have tested whether the task increases career exploration or proactive behavior. They have not tested whether the task changes the quality of the thinking that accompanies exploration. That omission is the gap the present study addresses.

### 1.3 The exploration paradox

Career exploration is the process of gathering and evaluating information about the self and about the occupational environment (Stumpf et al., 1983). The default assumption in the field is that more exploration produces better decisions.

The evidence does not support that assumption cleanly. A comprehensive meta-analysis of students' career exploration by Kleine et al. (2021) documented substantial heterogeneity in the associations between career exploration and decision-related outcomes. Similar heterogeneity appeared in the review of Jiang et al. (2019) and in the meta-analytic work of Lee et al. (2023) on personality predictors of career exploration. The personality meta-analysis showed that self-exploration and environmental exploration related differently to trait predictors. This finding is consistent with the possibility that the construct itself is heterogeneous rather than uniformly adaptive. Recent longitudinal work by Morgan et al. (2024) further demonstrated that career exploration behaviors relate to career decision-making through self-regulated learning processes. The relationship between exploration and decision outcomes is therefore neither direct nor simple.

The dominant response to heterogeneity has been to search for moderators. Investigators have proposed that exploration helps only when self-efficacy is high. Others have proposed that it helps only when social support is available or only when tolerance of ambiguity is sufficient (Xu and Tracey, 2015). This strategy has produced many published models. It has not resolved the underlying inconsistency.

The moderator strategy rests on an assumption that is rarely examined. It assumes that career exploration is a homogeneous construct whose effect varies by context. An alternative explanation is that the construct itself is heterogeneous. Two students may report identical amounts of exploration while engaging in qualitatively different processes. One student gathers information and updates a working hypothesis. The other student cycles through the same doubts without resolution. Existing exploration measures cannot separate these two students.

### 1.4 Reflective and ruminative career exploration

Identity development research encountered and solved an equivalent problem. Exploration in the identity literature had likewise shown inconsistent associations with adjustment. Luyckx et al. (2008) resolved this by adding a fifth dimension to the four-dimensional model of identity formation. They labeled it ruminative exploration. Ruminative exploration is repetitive and indecisive self-questioning that fails to converge on commitment. Their findings were clear. Ruminative exploration correlated positively with distress and with self-rumination. The two reflective forms of exploration were unrelated to wellbeing and correlated positively with self-reflection.

Recent validation studies have confirmed the cross-cultural robustness of this distinction. Rivnyák et al. (2022) validated the Dimensions of Identity Development Scale including its ruminative exploration dimension in a Hungarian adolescent sample. Sánchez-Queija et al. (2024) validated the instrument in a Spanish university sample and found that ruminative exploration had a stronger effect on adjustment than the reflective exploration dimensions. Den Boer et al. (2026) conducted a five-wave longitudinal study of Dutch young adults transitioning from university to work and found that ruminative exploration decreased over time as identity commitments solidified.

The distinction maps onto a broader literature on repetitive thought. Nolen-Hoeksema et al. (2008) distinguished rumination from reflection on the basis of whether repetitive thought is abstract and evaluative or concrete and problem-focused. Trapnell and Campbell (1999) showed that self-rumination and self-reflection load on separate factors and relate to different personality dimensions.

Ruminative career exploration is nevertheless not simply domain-general rumination observed in a career context, and three conceptual differences justify treating it as a separate construct. The first is the domain over which the disposition operates. Trait self-rumination is a stable tendency toward abstract evaluative self-focus that generalizes across situations (Trapnell and Campbell, 1999), whereas ruminative career exploration is defined over a single content domain and a single developmental task; a student may cycle unproductively through occupational alternatives without any general tendency to ruminate, and the converse also holds. The second is the object of the repetitive thought. General rumination is directed at the self and at the causes and consequences of one's own affective states, whereas ruminative career exploration is directed at an unresolved decision and is therefore identified by its failure to converge rather than by its affective focus, which is the property that distinguishes unconstructive from constructive repetitive thought in contemporary accounts (Watkins and Roberts, 2020). The third is expected malleability. A dispositional tendency should be relatively stable across a 4-week interval, whereas exploration of an unresolved decision should respond to anything that supplies a criterion for closing the decision, which is precisely what the present trial tests. These three differences generate an empirical prediction, namely that the two constructs should not be interchangeable in a measurement model, and that prediction is tested in Section 2.5.3.

To our knowledge this distinction has not previously been transferred to the vocational domain in the form of a domain-specific measure evaluated within an intervention trial, although we state the claim as a bounded one rather than as an exhaustive survey of the literature. Two considerations explain why the gap has persisted. First, the Dimensions of Identity Development Scale of Luyckx et al. (2008) was written for the content domain of general future plans rather than for careers. Its items ask about life direction in the abstract. Second, the closest vocational instrument measures something different. The Vocational Identity Status Assessment of Porfeli et al. (2011) includes a career reconsideration factor with a self-doubt subscale. Career reconsideration concerns the revisiting of a commitment that has already been formed. Ruminative career exploration concerns the quality of the exploration process itself and does not presuppose an existing commitment. The contribution of the present study is therefore bounded in a corresponding way: it lies in supplying a career-domain measure of the process, in separating that measure from the neighboring constructs, and in testing it as a mechanism within a randomized trial, rather than in introducing the reflective-ruminative distinction itself, which is established in the identity literature.

Discriminant validity therefore requires explicit attention. Ruminative career exploration must be separable from three neighboring constructs. It must be separable from general indecisiveness, which is a trait-level disposition captured within the lack of readiness category of Gati et al. (1996). It must be separable from trait rumination, which is domain-general (Trapnell and Campbell, 1999). It must be separable from career reconsideration (Porfeli et al., 2011). We test all three separations in the present study.

### 1.5 The present study

Three theoretical frameworks are drawn on in the preceding sections and it is worth stating how they stand to one another, because they are not competing accounts of the same phenomenon. Identity development theory, and specifically the dual-cycle model of Luyckx et al. (2008), is the primary framework of the present study. The central move of the paper, which is to separate the quality of exploration from its quantity, originates in that model, and the construct we adapt and test as the mechanism is defined within it. Proactive motivation theory (Parker et al., 2010) is the framework within which future work self salience was introduced and within which existing intervention work has been interpreted (Strauss et al., 2012; Taber and Blankemeyer, 2015; Talluri et al., 2025); it is not displaced here but is used to generate the rival account that the trial tests, since it is that framework which predicts an additive, exploration-increasing pathway. The dual-process theory of career decision-making (Xu, 2023a,b) enters only at the point of interpretation, as an explanation of why the readiness and consistency dimensions rather than the information dimension should be responsive. The design therefore uses the second framework to specify what a subtractive account must be distinguished from, and the third to interpret the pattern that the first predicts.

We contrast two mechanisms by which a future work self writing intervention might reduce career decision-making difficulties. The additive mechanism holds that the intervention works by increasing reflective career exploration. Clarifying a hoped-for identity raises the value of information and therefore motivates further search. The subtractive mechanism holds that the intervention works by decreasing ruminative career exploration. Clarifying a hoped-for identity supplies an organizing standard that terminates unresolved cycling.

The reasoning behind the subtractive account can be stated in three steps. Ruminative exploration is characterized by the absence of a criterion against which alternatives can be closed off: repetitive comparison persists because nothing determines when comparison is complete, which is what distinguishes it from information search that terminates when the relevant information has been obtained (Luyckx et al., 2008; Watkins and Roberts, 2020). A future work self is a representation of an identity rather than of an outcome (Strauss et al., 2012), and once it is sufficiently elaborated and accessible it can serve as such a criterion, because an alternative can then be evaluated as fitting or not fitting that identity rather than only as better or worse than some other alternative. The writing task is designed to act on both components of salience: the four sessions require concrete elaboration of the future self, of its working day, of its capabilities and relationships and of one connecting step, and repeated retrieval across 4 weeks increases accessibility. The prediction that follows is subtractive rather than additive, because supplying a criterion terminates cycling without necessarily increasing the volume of information that is sought. The task also shares with the best possible self paradigm (King, 2001; Sheldon and Lyubomirsky, 2006) the requirement of concrete and problem-focused rather than abstract and evaluative processing, which is the contrast on which the rumination literature turns (Nolen-Hoeksema et al., 2008; Watkins and Roberts, 2020).

The two mechanisms make different predictions across the taxonomy of Gati et al. (1996). The additive mechanism predicts effects concentrated on lack of information. The subtractive mechanism predicts effects concentrated on lack of readiness and on inconsistent information. The rationale for that allocation follows from the content of the three categories. Lack of readiness comprises low motivation, general indecisiveness and dysfunctional beliefs, and the indecisiveness component in particular is sustained by unresolved repetitive comparison, so a manipulation that interrupts such comparison should act on it directly; consistent with this, readiness-related difficulties are relatively trait-like and resistant to informational input (Saka et al., 2008), which is why an information-supplying intervention would not be expected to move them. Inconsistent information comprises internal and external conflict among alternatives, and a criterion that determines which alternatives fit an intended identity resolves conflict by ranking the alternatives, without any of the conflicting information itself having to change. Lack of information, by contrast, reflects a genuine deficit in what the student knows about the decision process, the self, occupations and information sources; no reorganization of existing content can supply content that is absent, so the subtractive account predicts no effect on this dimension whereas the additive account predicts its largest effect there. The two mechanisms are therefore empirically distinguishable rather than merely verbal alternatives. Figure 1 presents the competing models. Figure 1 is a statement of the two competing sets of predictions and contains no results. The plus and minus signs denote the direction of the association that each account predicts, and the dashed paths denote associations that the corresponding account does not predict. The observed pattern of effects is reported separately in Section 3 and is not anticipated in the figure.

**Figure 1:**
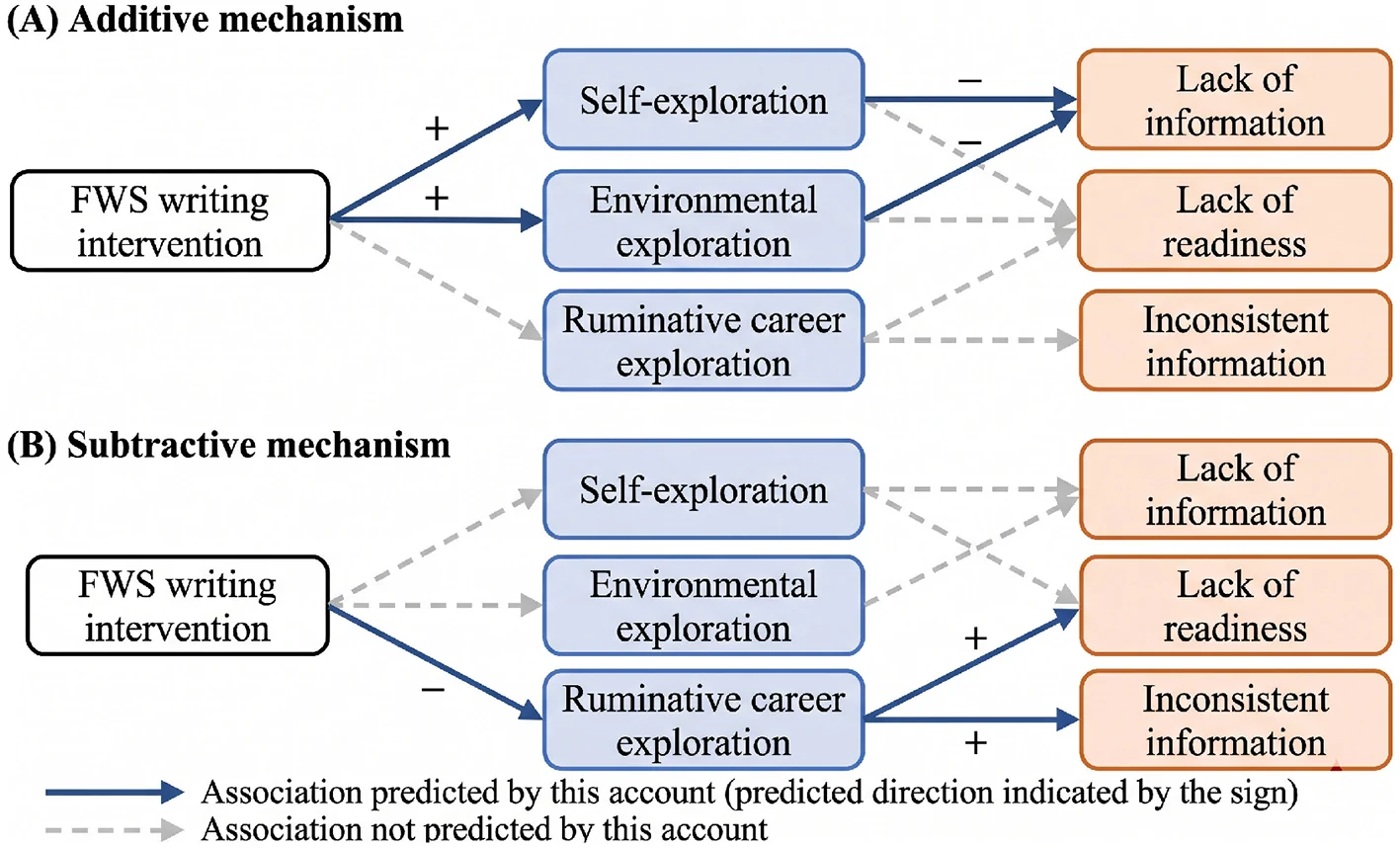
Competing additive and subtractive mechanism models linking the future work self writing intervention to the three dimensions of career decision-making difficulties through reflective and ruminative career exploration. Solid arrows denote associations predicted by the account, with the predicted direction given by the sign; dashed arrows denote associations the account does not predict. The figure presents competing predictions only; no results are displayed. **(A)** Additive mechanism. **(B)** Subtractive mechanism.

We tested five hypotheses.

H1. Relative to both control conditions the future work self writing condition increases future work self salience from baseline to post-intervention.

H2. Relative to both control conditions the future work self writing condition decreases ruminative career exploration.

H3. Relative to both control conditions the future work self writing condition decreases career decision-making difficulties. The effect is predicted for the lack of readiness and inconsistent information dimensions and is not predicted for the lack of information dimension.

H4. Change in ruminative career exploration mediates the intervention effect on career decision-making difficulties. Change in reflective career exploration does not mediate this effect. The two indirect effects differ significantly from each other.

H5. Intervention effects extend to employment outcomes measured at the 3-month follow-up. This hypothesis is exploratory.

H1 to H4 are confirmatory and were registered before data collection, together with the design, the three conditions, the assessment schedule and the outcome instruments; H5 was registered as exploratory. The three dimensions of the taxonomy are analyzed separately under H3, and the parallel multiple mediator model is the analysis specified by H4, so both follow from the registered hypotheses; what the registration does not itemize is the predicted direction of individual paths within the mediation models. The elements of the present version that were added during peer review, and that are therefore *post-hoc*, are identified in Section 2.1. The corresponding designation of primary, secondary and exploratory outcomes is stated in Section 2.5.7.

## 2 Materials and methods

### 2.1 Design, registration, and ethics

We conducted a three-arm parallel-group randomized controlled trial with allocation in a 1:1:1 ratio. Assessments occurred at four points. These were baseline (T1) and immediately after the 4-week intervention (T2) and 1-month follow-up (T3) and 3-month follow-up (T4).

The trial was prospectively registered at the Chinese Clinical Trial Registry (ChiCTR2500074449). Registration preceded the first participant assessment. All participants provided written informed consent before enrolment.

We report the scope of the registration explicitly so that readers can distinguish pre-specified from *post-hoc* elements. The registered record specifies the design, the three conditions, the assessment schedule and the outcome instruments listed in Section 2.5, together with the five hypotheses stated in Section 1.5. The separate analysis of the three difficulty dimensions follows from H3 as registered and the parallel multiple mediator model is the analysis specified by H4, so neither is a *post-hoc* addition; the registration does not, however, itemize predictions for individual paths within the mediation models. The designation of primary, secondary and exploratory outcomes described in Section 2.5.7, the equivalence tests described in Section 2.7 and the multiplicity corrections applied to the employment indicators were added during peer review and are identified as *post-hoc* throughout. No outcome was added, removed or re-specified after the data were examined.

The study protocol was approved by the Ethics Committee for Humanities and Social Sciences Research of Anhui University of Science and Technology (approval number AUST-HSS-IRB-2025-058). Reporting follows the CONSORT 2010 statement (Schulz et al., 2010). The completed CONSORT checklist appears in Supplementary Table S1. This study involved human participants throughout. Ethics approval, informed consent and the human-participant status of the trial are stated in the Ethics Statement at the end of this article, and the corresponding declaration in the submission system has been corrected accordingly.

### 2.2 Participants and recruitment

Participants were final-year undergraduate and postgraduate students recruited from 4 universities in Eastern China. The sampling frame deliberately spanned institutional tiers. It included 2 universities in the national key construction programme (Project 211/Double First Class) and 2 regional universities. This design addresses the representativeness limitation common to single-site convenience samples.

Recruitment was conducted at the four participating universities in the period immediately preceding the baseline assessment in early September, through the career services offices and through announcements posted on institutional student-information channels. No classroom-based or social-media recruitment was used. Students who expressed interest completed an online eligibility screen. A total of 612 students were screened. Of these, 126 were not randomized: 74 did not meet the inclusion criteria, 38 declined to participate after reading the information sheet and 14 were excluded for other reasons, including duplicate registrations and withdrawal of contact before allocation. The remaining 486 students were randomized.

The number of students screened and randomized at each site was as follows: key university 1, 178 screened and 141 randomized; key university 2, 152 screened and 102 randomized; regional university 1, 156 screened and 132 randomized; and regional university 2, 126 screened and 111 randomized. Because randomization was stratified by university, allocation was closely balanced across the three arms within every site, departing from exact equality only where the final block within a stratum was incomplete; the resulting site by arm cell sizes are reported in Supplementary Table S6.

Recruitment continued until the pre-specified target of 488 participants was approached and was closed when the autumn recruitment season opened, at which point 486 eligible students had been enrolled. Participant flow after randomization, including the number analyzed at each wave in each arm, is reported in Section 3.1.

Inclusion criteria were enrolment in the final year of study, expected graduation within the current academic year and provision of informed consent. Exclusion criteria were current enrolment in another career intervention study and prior completion of a structured future work self writing exercise.

The required sample size was estimated with G*Power 3.1 (Faul et al., 2007). The target analysis was the group by time interaction in a repeated measures design with three groups and four measurement occasions. We assumed a small to medium effect of *f* = 0.25 with power of 0.80 and α = 0.05. We further assumed a correlation among repeated measures of 0.60. The required total sample was 390. We inflated this figure by 25 percent to accommodate anticipated attrition. The final recruitment target was 488 participants. With the achieved sample of 162 participants per arm, the smallest between-arm difference detectable with 80 percent power at α = 0.05 corresponds to *d* = 0.31. This value is used in Section 2.7 to define the smallest effect size of interest for the equivalence tests.

### 2.3 Randomization and blinding

The allocation sequence was generated by an independent statistician using a computerized random number generator in R. Randomization was stratified by university and by degree level. Permuted blocks of size six were used within strata. Allocation was concealed from the recruiting researchers through sequentially numbered opaque sealed envelopes.

We state the three implementation roles separately because they were held by different people. The allocation sequence was generated by an independent statistician who had no other role in the trial and no contact with participants, and who did not take part in recruitment, delivery or analysis. Participants were enrolled by research assistants based at the career services office of each participating university, who administered the online eligibility screen, obtained written informed consent and had no access to the allocation sequence at any point. Participants were assigned to conditions by a research coordinator who was not involved in recruitment or in outcome assessment, who opened the sequentially numbered opaque sealed envelopes in sequence only after the baseline assessment of the participant concerned had been completed and recorded, so that allocation could not influence baseline measurement.

Participants could not be blinded to condition because the writing tasks differ in visible content. Participants were informed only that the study compared alternative reflective writing exercises. Outcome assessment relied on self-report and could not be blinded at the participant level. Data analysts were blinded to condition labels until the primary analyses were complete. Condition labels were replaced by arbitrary codes in the analysis dataset.

### 2.4 Interventions

All three conditions were delivered through the same online platform. All conditions used the same schedule and the same reminder protocol.

#### 2.4.1 Future work self writing condition

Participants completed 4 weekly writing sessions of approximately 20 min. Session one asked participants to imagine themselves in their working life several years ahead and to describe that self in concrete detail. Session two asked participants to describe a typical working day for that future self. Session three asked participants to identify the capabilities and relationships that future self would possess. Session four asked participants to describe one step connecting the present self to that future self. The instructions were adapted from the future work self paradigm of Strauss et al. (2012) and from the best possible self writing paradigm (King, 2001; Sheldon and Lyubomirsky, 2006). Full instructions appear in Supplementary material S5.

#### 2.4.2 Active control condition

Participants completed 4 weekly writing sessions of approximately 20 min on general future planning unrelated to occupational identity. Topics included planning a journey and planning a shared living arrangement. Further topics were planning a personal learning project and planning a community activity. The active control was matched to the intervention on four features. These features were session duration and session frequency. They also included response format and expected word count. This matching allows the effect of writing about a future work self to be separated from the effect of structured writing in general.

#### 2.4.3 Waitlist control condition

Participants completed assessments only. They received the full future work self writing programme after the 3-month follow-up assessment. This arrangement satisfies the ethical requirement that no participant be denied a potentially beneficial intervention.

#### 2.4.4 Fidelity and adherence

Adherence was operationalized as the number of sessions completed and the word count of each submission. Two independent raters coded a random 20 percent of the participants who received a writing condition, coding every submission made by each sampled participant, for on-topic content using a rubric reported in Supplementary Table S2. Interrater agreement is reported in Section 3.4. A manipulation check assessed whether the future work self writing condition produced the intended increase in future work self salience.

### 2.5 Measures

All instruments were administered in Chinese. Instruments without an established Chinese version were translated and back-translated following the procedure of Brislin (1970). Table 1 summarizes all measures with source and item count. It also reports response format and internal consistency in the present sample.

**Table 1:** Overview of measures with source, item count, response format and internal consistency.

Construct	Source	Items	Scale	ω
Future work self salience	Strauss et al., 2012	5	5-pt	0.89
Self-exploration	Stumpf et al., 1983	5	5-pt	0.84
Environmental exploration	Stumpf et al., 1983	4	5-pt	0.82
Ruminative career exploration	Adapted from Luyckx et al., 2008	7	5-pt	0.91
Lack of readiness	Gati et al., 1996	10	9-pt	0.83
Lack of information	Gati et al., 1996	12	9-pt	0.90
Inconsistent information	Gati et al., 1996	10	9-pt	0.87
Trait self-rumination	Trapnell and Campbell, 1999	12	5-pt	0.88
Career reconsideration	Porfeli et al., 2011	6	5-pt	0.85

#### 2.5.1 Future work self salience

Future work self salience was measured with the five-item scale of Strauss et al. (2012). Items are rated on a five-point scale. A sample item asks how easily the participant can imagine the self they hope to become at work. Higher scores indicate greater salience.

#### 2.5.2 Reflective career exploration

Reflective career exploration was measured with the self-exploration and environmental exploration subscales of the Career Exploration Survey (Stumpf et al., 1983). Items are rated on a five-point frequency scale. The two subscales were modeled separately.

#### 2.5.3 Ruminative career exploration

Ruminative career exploration was measured with a scale adapted to the career domain from the ruminative exploration subscale of the Dimensions of Identity Development Scale (Luyckx et al., 2008). Adaptation proceeded in four steps and followed the sequence of item generation, content validation, item reduction and psychometric evaluation recommended for scale development in the behavioral sciences (Boateng et al., 2018). First, the original items were rewritten to reference occupational rather than general future content. Second, three vocational psychology researchers reviewed item content for domain relevance. The three reviewers held doctoral degrees in vocational or organizational psychology and each had published peer-reviewed work on career development. They rated every candidate item independently on a four-point scale for relevance to the target construct and for clarity of wording, and they were asked to flag any item that could be read as referring to general life planning, to a settled occupational commitment or to domain-general rumination rather than to the career exploration process itself. Items rated as not relevant or as ambiguous by any reviewer were rewritten and re-rated until agreement was reached. The reviewers were not involved in data collection or analysis. Third, translation and back-translation followed Brislin (1970). Fourth, the resulting item pool was administered to an independent pilot sample of 185 final-year students who did not participate in the trial.

Exploratory factor analysis in the pilot sample is reported in Table 2. We report the procedure in full. The pilot sample of 185 respondents corresponds to a respondent-to-item ratio of approximately 23:1, which exceeds the ratio of 10:1 commonly recommended for factor analysis (Boateng et al., 2018). Factorability of the correlation matrix was checked before extraction. The Kaiser-Meyer-Olkin measure of sampling adequacy was 0.91, and Bartlett's test of sphericity was significant, χ^2^(28) = 987.64, *p* < 0.001, so the matrix was suitable for factor analysis. Factors were extracted by principal axis factoring, and the number of factors to retain was determined by eigenvalues greater than one together with inspection of the scree plot. A single factor was retained, so no rotation was applied. The extracted factor accounted for 62.3 percent of the item variance with an eigenvalue of 4.98; communalities ranged from 0.53 to 0.71 and corrected item-total correlations from 0.67 to 0.78 across the eight candidate items, indicating that the pool was factorable and internally coherent. An initial pool of eight items was tested. Item 8 was dropped because its factor loading (0.73) fell below the a priori retention criterion of 0.75. The final retained scale comprised seven items, with corrected item-total correlations from 0.69 to 0.78. The full item pool with Chinese translations appears in Supplementary Table S4. Confirmatory factor analysis in the trial baseline sample is reported in Table 3 together with evidence for discriminant validity. The confirmatory model was estimated by maximum likelihood, with full information maximum likelihood for missing data. Model fit was judged against the criteria of Hu and Bentler (1999), and discriminant validity was evaluated by comparing a two-factor solution with a merged one-factor solution for each neighboring construct, so that a substantial deterioration in fit for the merged solution indicates that the two constructs are not interchangeable. Discriminant validity was evaluated against three neighboring constructs. These were general indecisiveness from the lack of readiness category (Gati et al., 1996), trait self-rumination (Trapnell and Campbell, 1999) and career reconsideration (Porfeli et al., 2011). Incremental validity over reflective career exploration is reported in the same table.

**Table 2:** Exploratory factor analysis of the adapted ruminative career exploration item pool in the pilot sample (*n* = 185). Item 8 was dropped (loading below 0.75 retention criterion).

Item	Loading	*h* ^2^	*r*_*it*_
1. I keep asking myself whether my career plan is right	0.82	0.67	0.76
2. I worry repeatedly about what career suits me	0.79	0.62	0.73
3. I doubt my career choices without reaching a conclusion	0.84	0.71	0.78
4. I go over and over the same career concerns in my mind	0.81	0.66	0.75
5. I feel stuck cycling through the same occupational doubts	0.77	0.59	0.71
6. I cannot stop thinking about career problems I cannot solve	0.80	0.64	0.74
7. I keep comparing career options without making progress	0.75	0.56	0.69
8. I revisit the same career worries day after day^†^	0.73	0.53	0.67
Eigenvalue	4.98		
Variance explained (%)	62.3		

^†^Dropped from final scale.

Extraction method: principal axis factoring. Factor retention rule: eigenvalues greater.

Than one together with inspection of the scree plot. A single factor was retained and no rotation was applied. Kaiser-Meyer-Olkin measure of sampling adequacy = 0.91;

Bartlett's test of sphericity χ^2^(28) = 987.64, *p* < 0.001.

**Table 3:** Confirmatory factor analysis and discriminant validity evidence for ruminative career exploration (*N* = 486).

Model	χ^2^	df	CFI	RMSEA	SRMR
One-factor RCE (7 items)	28.41	14	0.976	0.046	0.029
Two-factor with general indecisiveness	72.83	26	0.969	0.061	0.037
Merged with general indecisiveness	198.52	27	0.885	0.115	0.078
Two-factor with trait self-rumination	114.67	53	0.964	0.049	0.035
Merged with trait self-rumination	312.40	54	0.867	0.100	0.082
Two-factor with career reconsideration	89.24	34	0.971	0.058	0.034
Merged with career reconsideration	246.31	35	0.891	0.112	0.076
AVE	0.63				
CR	0.92				
Incremental Δ*R*^2^ over reflective exploration	0.09, *p* < 0.001				

All models were estimated by maximum likelihood with full information maximum likelihood for missing data. Each merged model constrains the two constructs to load on a single factor; a substantial deterioration in fit relative to the corresponding two-factor model indicates that the constructs are not interchangeable. In the two-factor and merged models each neighboring construct was represented by parceled indicators, so the degrees of freedom are smaller than the item counts in Table 1 imply.

All models were estimated in the trial baseline sample, which is the same sample used for the substantive analyses reported in Section 3; the indices are therefore not cross-validated.

Two features of this evidence require explicit qualification. First, the confirmatory factor analysis and the discriminant validity comparisons were estimated in the trial baseline sample, which is the same sample that provides the substantive findings reported in Section 3, rather than in an independent validation sample. These indices are therefore not cross-validated and are likely to be more favorable than would be obtained in a fresh sample. Second, the trial did not include an established measure of distress or negative affect, so no direct convergent validity coefficient against such a measure is available. The evidence bearing on the affective character of the construct is therefore indirect and consists of the pattern of associations reported in Section 3.5, in which ruminative career exploration is moderately and positively associated with all three difficulty dimensions and negatively associated with future work self salience, while its associations with the two reflective exploration subscales are close to zero. Both points are treated as limitations in Section 4.4 and as priorities for the validation study described in Section 4.5.

#### 2.5.4 Career decision-making difficulties

Career decision-making difficulties were measured with the Career Decision-Making Difficulties Questionnaire (Gati et al., 1996). The three superordinate dimensions were scored and modeled separately in line with the evidence for their differential behavior (Saka et al., 2008). A total score is reported for descriptive comparison with prior literature but is not used in the primary analyses.

#### 2.5.5 Employment outcomes

Employment outcomes were recorded at the 3-month follow-up. Four indicators were collected. These were signed employment agreement status and number of offers received. They also included satisfaction with employment quality and perceived match between the position and the field of study. All four indicators were single-item self-reports and are treated as exploratory throughout. Descriptive values by condition, effect estimates with confidence intervals and both uncorrected and corrected p values are reported in Section 3.9.

#### 2.5.6 Covariates

Covariates were sex, degree level and disciplinary field. Institutional tier and prior completion of a formal career course were also recorded. Baseline values of each outcome were included as covariates in the corresponding models.

#### 2.5.7 Designation of primary, secondary and exploratory outcomes

The trial collected a large number of indicators and the number of statistical tests reported is correspondingly large, so we state the outcome hierarchy explicitly. The primary outcomes are ruminative career exploration, which is the mediator specified by the subtractive account, and the lack of readiness dimension of career decision-making difficulties, which is the difficulty dimension on which that account predicts the largest effect. The secondary outcomes are the self-exploration and environmental exploration subscales, the lack of information and inconsistent information dimensions, and future work self salience, which also serves as the manipulation check for H1. The mediation model reported in Section 3.8 is a pre-specified mechanistic analysis and is interpreted as secondary. The four employment indicators are exploratory, consistent with the exploratory status already assigned to H5. This hierarchy follows the order in which the hypotheses are stated in Section 1.5 and was fixed before the corrections described below were applied; it was not used to select which results to report, and results for every outcome collected are reported in Section 3 irrespective of significance.

### 2.6 Procedure

Figure 2 presents the study timeline aligned with the Chinese recruitment calendar. Baseline assessment occurred in early September before the autumn recruitment season opened. The intervention ran across four consecutive weeks in September. Post-intervention assessment occurred in early October. The 1-month follow-up occurred in November at the close of autumn recruitment. The 3-month follow-up occurred in January. Each assessment took approximately 20 min. Participants received modest compensation at each wave.

**Figure 2:**
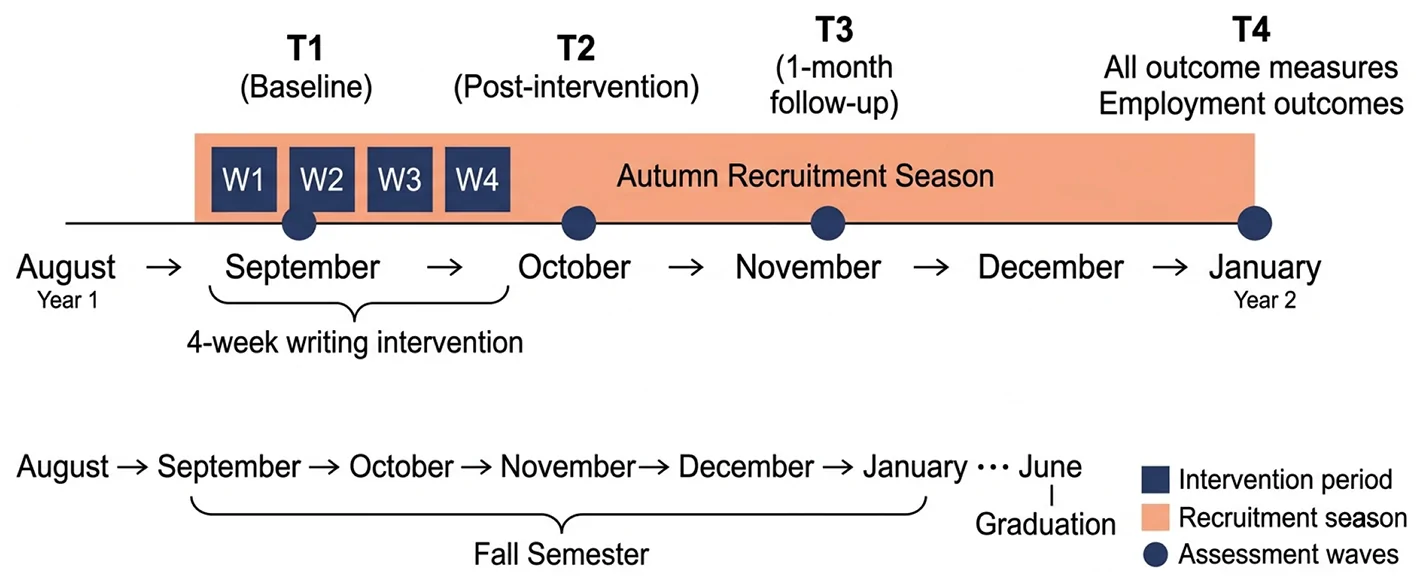
Study timeline aligned with the Chinese graduate recruitment calendar showing the four assessment waves and the intervention period.

### 2.7 Analytic strategy

All primary analyses followed the intention-to-treat principle. Participants were analyzed in the condition to which they were randomized regardless of adherence. Missing data were handled with full information maximum likelihood.

We state the assumption on which that procedure rests, because the original description was open to being read as a stronger claim than the data can support. Full information maximum likelihood yields consistent estimates when data are missing at random, which is a weaker condition than missing completely at random, and the missing at random assumption is not testable against the observed data (Graham, 2009). Attrition was therefore examined in two ways that bear on the plausibility of the assumption rather than establishing it. First, the test of Little (1988) was computed and is reported in Section 3.1 for completeness; because it has limited power and because failure to reject a null hypothesis is not evidence for it, a non-significant result is not treated here as demonstrating that data are missing completely at random. Second, logistic regressions were estimated at each follow-up wave predicting dropout from all baseline demographic characteristics and from the baseline value of every outcome, with condition entered as a predictor so that differential attrition across arms could be detected; the full results are reported in Supplementary Table S7. Variables associated with missingness in these models were retained in the analysis models, which is the condition under which the missing at random assumption becomes more defensible (Graham, 2009). Data that are missing not at random cannot be excluded by any of these procedures, and this residual possibility is recorded as a limitation in Section 4.4.

Longitudinal measurement invariance was tested across the four waves for every latent outcome. We tested configural, metric and scalar invariance in sequence. Invariance was evaluated with the change in comparative fit index using the criterion of Cheung and Rensvold (2002). Model fit was evaluated against the criteria of Hu and Bentler (1999). Results for all seven latent outcomes are reported in Section 3.3.

Primary analyses used latent growth curve modeling with condition entered as dummy-coded predictors of the slope factor. Separate models were estimated for future work self salience, for each exploration variable and for each dimension of career decision-making difficulties. This approach is consistent with recent methodological guidance on using latent growth curve models to evaluate intervention effects in randomized controlled trials (Grimm et al., 2016).

We specify these models in full. Each model contained a latent intercept factor and a latent linear slope factor defined by the four repeated measurements of the outcome. Loadings on the intercept factor were fixed to one at every wave. Loadings on the slope factor were fixed to the elapsed time from baseline in months, that is 0, 1, 2, and 4 for T1 to T4 respectively, so that the intercept is centered at baseline and the slope represents the rate of change per month. Residual variances of the repeated indicators were estimated freely and were not constrained to equality across waves. Condition was represented by two dummy variables regressed on the slope factor. Each of the two contrasts reported in Section 3.6 was obtained by re-estimating the model with the corresponding condition as the reference category. The unstandardized coefficient *b* reported in Section 3.6 is the model-implied between-condition difference at the final assessment (T4), obtained by multiplying the difference in the slope factor by the T4 time loading of 4 and expressed in the original metric of the outcome. Because the intercept is centered at baseline and the baseline value of the outcome is captured by the intercept factor, no additional baseline adjustment was applied to the same outcome; the covariates listed in Section 2.5.6 were grand-mean centered before entry and were regressed on both the intercept and the slope factors. Effect sizes were computed as *d* = *b* divided by the pooled baseline standard deviation of the outcome, and confidence intervals for *d* were obtained by applying the same transformation to the limits of the interval for *b*; every effect size reported in this article is therefore accompanied by an interval estimate. Fit indices for every growth model are reported in Section 3.3.

Two features of the design have implications for the standard errors. Randomization was stratified by university and by degree level, so allocation is balanced within each university and the treatment contrasts are not confounded with university-level differences. Nevertheless the four universities constitute clusters, and students within a university may be more similar to one another than to students elsewhere. We did not model this clustering with a random intercept for university. With only four clusters the between-cluster variance component is identified by very little information and cluster-robust standard errors are correspondingly unstable, so the resulting inference can be less reliable than the inference it is intended to correct. We note that this pessimistic assessment of likelihood-based estimation with few upper-level units has itself been qualified: Elff et al. (2021) show that the bias and the anti-conservative inference reported in earlier simulation work are largely removed when restricted maximum likelihood is combined with a Kenward-Roger type adjustment. That work nevertheless concerns designs with more upper-level units than the four available here, and we did not regard four clusters as sufficient to support either a random intercept or a cluster-robust correction. Institutional tier, which is the design variable that distinguishes the two categories of participating universities, was included as a covariate in every model. We nonetheless treat the omission of an explicit cluster term as a limitation of the analysis and address it in Section 4.4.

Multiplicity was handled within families of outcomes rather than across the whole set of tests. Three families were defined: the three dimensions of career decision-making difficulties, the two reflective career exploration subscales, and the four employment indicators. A Bonferroni correction was applied within each family, and both uncorrected and corrected p values are reported for each. Corrections within the employment family were added in response to peer review and are identified as *post-hoc* in Section 3.9.

Non-significant results were not interpreted as evidence of the absence of an effect without an explicit test of that claim. For the reflective career exploration outcomes we therefore conducted equivalence tests using the two one-sided tests procedure (Lakens et al., 2018). The smallest effect size of interest was set at *d* = 0.30. This is the value of *d* = 0.31 reported in Section 2.2, rounded to one decimal place. It is the smallest between-arm effect that 162 participants per arm can detect with 80 percent power at α = 0.05. Effects smaller than this bound could not have been detected reliably by this trial and are treated as not distinguishable from zero. Equivalence tests were computed from the latent growth model estimates and their standard errors expressed in the effect size metric, and are reported in Section 3.6.

Mediation was tested in a parallel multiple mediator model estimated as a single structural equation model, separate from the latent growth curve models reported in Section 3.6. Latent change scores from baseline to post-intervention served as mediators. The outcome was the level of lack of readiness at the 1-month follow-up (T3), controlling for its baseline value, rather than the four-wave slope contrast used in the growth models. Indirect effects were estimated with bias-corrected bootstrap confidence intervals based on 10,000 resamples (Preacher and Hayes, 2008). We report a formal contrast of the two indirect effects. Concluding that one path is stronger requires the difference between indirect effects to be significant rather than merely one path reaching significance and the other failing to do so (Preacher and Hayes, 2008). Because the mediation model uses a different parameterisation from the growth models, the *a*-paths (condition → mediator change), the *b*-paths (mediator change → outcome) and the total effect are all estimated within the mediation model itself and need not equal the slope contrasts reported in Section 3.6. Any numerical correspondence between the two sets of estimates is a consequence of the data rather than a constraint of the specification.

The temporal structure of this model places the mediators, which are latent change scores from baseline to post-intervention, before the outcome, which is the level of lack of readiness at the 1-month follow-up controlling for its baseline value, and the exposure is randomized. Randomization of the exposure supports a causal interpretation of the total effect. It does not by itself license a causal interpretation of the indirect effects, because the mediator is not randomized and the estimates additionally require the absence of unmeasured confounding of the mediator-outcome relationship, an assumption that cannot be tested with the present data (Imai et al., 2010). No sensitivity analysis for violations of that assumption was conducted. We therefore describe the indirect effects as associations that are consistent with the subtractive account and reserve causal language for the randomized comparison of conditions.

Sensitivity analyses comprised per-protocol estimation, complier average causal effect estimation and correction for multiple comparisons across the three difficulty dimensions. Common method concerns were addressed through the procedural remedies described by Podsakoff et al. (2012). Analyses were conducted in Mplus 8.10 (Muthén & Muthén, Los Angeles, CA, USA) (Muthén and Muthén, 2017) and R 4.3.2 (R Foundation for Statistical Computing, Vienna, Austria) (R Core Team, 2024). The analysis code is available from the corresponding author upon reasonable request.

## 3 Results

### 3.1 Participant flow and attrition

Figure 3 presents the CONSORT flow diagram. Of 612 students screened, 486 met eligibility criteria and were randomized. Each condition received 162 participants. Retention was 94.0% at post-intervention, 89.7% at 1-month follow-up and 85.4% at 3-month follow-up.

**Figure 3:**
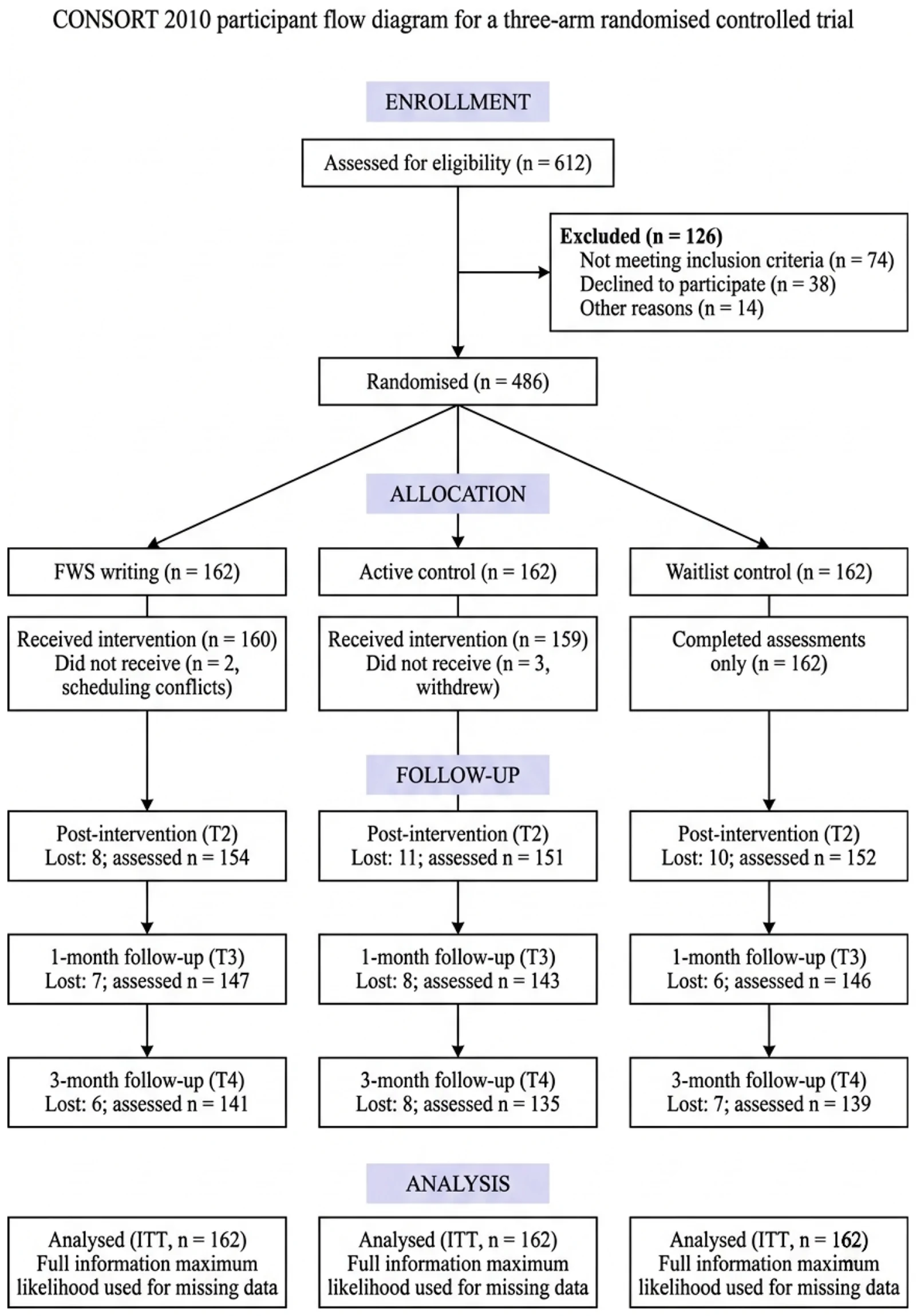
CONSORT flow diagram showing screening, randomization, allocation, follow-up and analysis for the three conditions. Each follow-up box reports the losses occurring in that arm only and the number assessed at that wave. The numbers of participants assessed are available-case numbers; the number analyzed under intention to treat is 162 in every arm at every wave because full information maximum likelihood was used.

Losses to follow-up are reported separately by arm. Two participants allocated to the future work self condition and three allocated to the active control condition did not begin the writing programme, because of scheduling conflicts and withdrawal respectively; all five were retained in the intention-to-treat analyses. Between randomization and the post-intervention assessment eight participants were lost in the future work self condition, 11 in the active control condition and 10 in the waitlist condition, leaving 154, 151, and 152 participants with post-intervention data. A further 7, 8, and 6 participants were lost before the 1-month follow-up, leaving 147, 143, and 146 participants, and a further 6, 8 and 7 before the 3-month follow-up, leaving 141, 135, and 139 participants. These figures correspond to the overall retention rates reported above, namely 457 of 486 at post-intervention, 436 of 486 at the 1-month follow-up and 415 of 486 at the 3-month follow-up. These are available-case numbers. Because full information maximum likelihood was used, all 486 randomized participants contributed to every intention-to-treat model and the number analyzed in each arm is 162 at every wave; the available-case numbers are reported so that the two quantities are not confused.

The missing-data analysis is reported next. The test of Little (1988) was non-significant, χ^2^ = 18.72, *df* = 14, *p* = 0.176. As stated in Section 2.7 this result is reported for completeness and is not interpreted as demonstrating that data are missing completely at random. Logistic regressions predicting dropout at each wave from condition, from all baseline demographic characteristics and from the baseline value of every outcome identified no significant predictor of attrition at any wave (all *p*>0.22), and attrition did not differ across the three arms in that model [χ^2^(2) = 1.04, *p* = 0.59 at the final wave]. Full model output is reported in Supplementary Table S7. Retention was high and non-differential, which limits though it does not eliminate the scope for bias, and the possibility that data are missing not at random cannot be excluded; this is recorded as the twelfth limitation in Section 4.4.

### 3.2 Baseline characteristics

Table 4 reports baseline demographic and outcome characteristics by condition. The three conditions did not differ on any baseline variable (all *p* > 0.10).

**Table 4:** Baseline characteristics by condition.

Characteristic	FWS	Active ctrl	Waitlist	Test
*n*	162	162	162	
Age, M (SD)	22.31 (1.14)	22.18 (1.21)	22.25 (1.09)	*F* = 0.47, *p* = 0.63
Female, *n* (%)	98 (60.5)	94 (58.0)	101 (62.3)	χ^2^ = 0.64, *p* = 0.73
Postgraduate, *n* (%)	41 (25.3)	39 (24.1)	43 (26.5)	χ^2^ = 0.27, *p* = 0.87
Key university, *n* (%)	82 (50.6)	80 (49.4)	81 (50.0)	χ^2^ = 0.05, *p* = 0.98
Prior career course, *n* (%)	68 (42.0)	71 (43.8)	65 (40.1)	χ^2^ = 0.47, *p* = 0.79
FWS salience, M (SD)	3.12 (0.78)	3.08 (0.81)	3.15 (0.74)	*F* = 0.30, *p* = 0.74
Ruminative expl., M (SD)	3.24 (0.82)	3.19 (0.79)	3.27 (0.85)	*F* = 0.38, *p* = 0.68
Self-exploration, M (SD)	2.87 (0.71)	2.91 (0.68)	2.84 (0.73)	*F* = 0.41, *p* = 0.66
Env. exploration, M (SD)	2.65 (0.76)	2.71 (0.74)	2.63 (0.79)	*F* = 0.44, *p* = 0.64
Lack of readiness, M (SD)	4.82 (1.34)	4.75 (1.29)	4.88 (1.38)	*F* = 0.37, *p* = 0.69
Lack of information, M (SD)	5.14 (1.41)	5.21 (1.45)	5.09 (1.37)	*F* = 0.26, *p* = 0.77
Inconsist. info., M (SD)	4.93 (1.31)	4.87 (1.27)	4.96 (1.35)	*F* = 0.18, *p* = 0.84

### 3.3 Measurement model and longitudinal invariance

Table 3 reports the confirmatory factor analysis and discriminant validity evidence for the ruminative career exploration scale. Table 5 reports the longitudinal measurement invariance tests. Invariance was tested for all seven latent outcomes and results for all seven are reported, in place of the subset presented in the original submission. Scalar invariance was supported for all latent outcomes (ΔCFI < 0.010 at each step).

**Table 5:** Longitudinal measurement invariance across the four assessment waves, for all seven latent outcomes.

Construct	Model	χ^2^	df	CFI	ΔCFI	RMSEA	Decision
Rum. expl.	Configural	142.38	98	0.981		0.031	Accept
	Metric	157.01	116	0.979	0.002	0.027	Accept
	Scalar	174.62	134	0.976	0.003	0.025	Accept
Self-expl.	Configural	68.21	48	0.984		0.029	Accept
	Metric	79.34	60	0.983	0.001	0.026	Accept
	Scalar	95.18	72	0.979	0.004	0.025	Accept
Lack of read.	Configural	189.42	146	0.974		0.025	Accept
	Metric	213.67	173	0.973	0.001	0.022	Accept
	Scalar	241.85	200	0.970	0.003	0.021	Accept
FWS salience	Configural	83.41	48	0.987		0.039	Accept
	Metric	93.64	60	0.985	0.002	0.034	Accept
	Scalar	110.03	72	0.981	0.004	0.033	Accept
Env. expl.	Configural	38.47	24	0.988		0.035	Accept
	Metric	47.91	33	0.987	0.001	0.031	Accept
	Scalar	62.34	42	0.983	0.004	0.032	Accept
Lack of info.	Configural	214.58	174	0.977		0.022	Accept
	Metric	247.31	207	0.975	0.002	0.020	Accept
	Scalar	283.94	240	0.972	0.003	0.019	Accept
Inconsist. info.	Configural	193.87	146	0.976		0.026	Accept
	Metric	217.39	173	0.974	0.002	0.023	Accept
	Scalar	246.95	200	0.971	0.003	0.022	Accept

Invariance was evaluated with the change in comparative fit index; ΔCFI below 0.010 at each step supports the corresponding level of invariance. Each step imposes 3(*k*−1) constraints, where *k* is the number of indicators, so constructs with the same number of indicators have the same degrees of freedom. The degrees of freedom for the metric and scalar models of ruminative career exploration were mis-transcribed in the original submission and are corrected here.

Fit indices for the latent growth models are reported in Table 6. CFI and SRMR met the criteria of Hu and Bentler (1999) for all seven models. RMSEA was at or below 0.06 for five models and marginally above it for ruminative career exploration (.061) and environmental exploration (0.066). Each growth model has only five degrees of freedom, and RMSEA is known to indicate poor fit too often in models with few degrees of freedom (Kenny et al., 2015), so we give more weight to CFI and SRMR in evaluating these models. The χ^2^ test was significant for five of the seven models, which is expected at this sample size and does not by itself indicate a substantively poor model. No model required *post-hoc* modification. The residual misfit has an identifiable source. The estimated trajectories reported in Sections 3.6 and 3.7 show that most of the change in the future work self condition occurs between baseline and the post-intervention assessment, after which the trajectories flatten. A linear slope is a parsimonious summary of that shape rather than an exact description of it, and with only five degrees of freedom available in each model this discrepancy is the most likely source of the significant χ^2^ values and of the two RMSEA values above .06. The direction of the consequence is worth stating explicitly: because a linear slope distributes a step-like change across all four occasions, the effect sizes reported in Section 3.6 are conservative relative to the immediate post-intervention difference between conditions rather than inflated by the specification. A piecewise growth model, or a model with freely estimated slope loadings, would follow the observed shape more closely; neither was specified in the analysis plan and neither is reported here.

**Table 6:** Fit of the unconditional linear growth curve model for each outcome (*N* = 486).

Outcome	χ^2^	df	*p*	CFI	RMSEA	SRMR
Ruminative career exploration	14.03	5	0.015	0.967	0.061	0.071
Self-exploration	11.81	5	0.038	0.972	0.053	0.054
Environmental exploration	15.57	5	0.008	0.959	0.066	0.036
Future work self salience	10.13	5	0.072	0.977	0.046	0.044
Lack of readiness	10.82	5	0.055	0.975	0.049	0.073
Lack of information	13.44	5	0.020	0.966	0.059	0.062
Inconsistent information	12.88	5	0.025	0.968	0.057	0.050

Fit indices are for the unconditional growth model of each outcome, estimated before condition and covariates were entered. With *N* = 486 the χ^2^ test is sensitive to trivial misspecification and is reported for completeness.

### 3.4 Fidelity, adherence, and manipulation check

Mean session completion was 3.71 of four sessions (*SD* = 0.52) in the future work self condition and 3.64 of four sessions (*SD* = 0.58) in the active control condition. Interrater agreement on on-topic coding was κ = 0.91. The manipulation check showed that the future work self condition produced a significantly larger increase in future work self salience from T1 to T2 than either the active control [*d* = 0.52, 95% CI [0.30, 0.74], *p* < 0.001] or the waitlist condition [*d* = 0.58, 95% CI [0.36, 0.80], *p* < 0.001]. This result supports H1. Figure 4 displays adherence and manipulation check results by condition.

**Figure 4:**
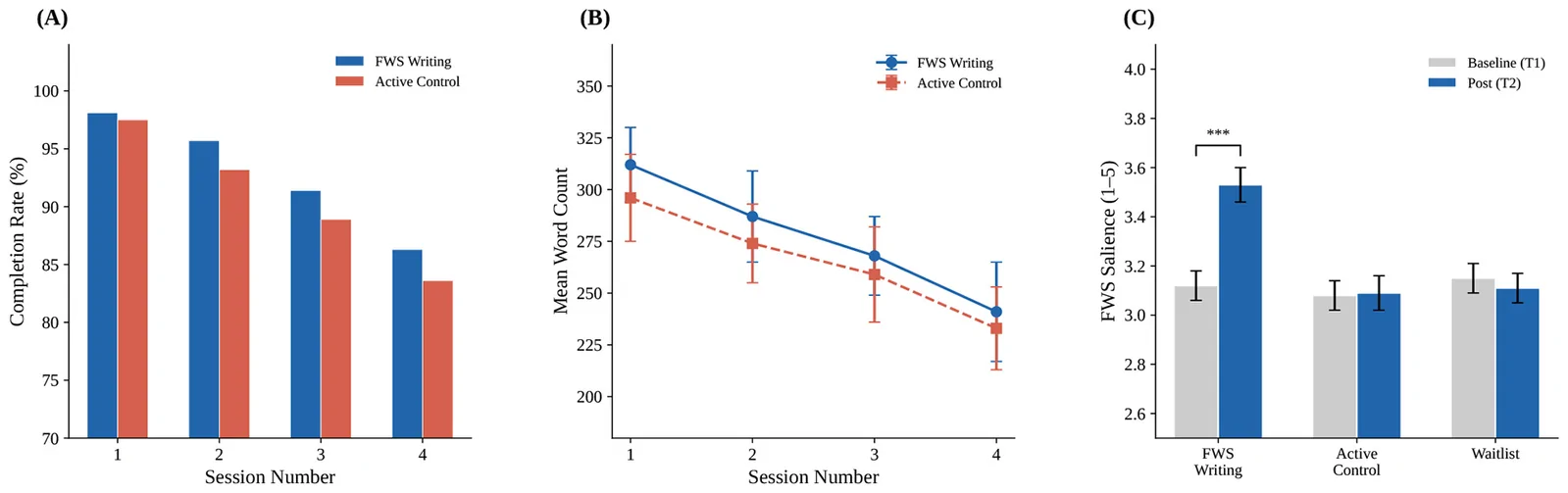
Intervention fidelity and manipulation check by condition. **(A)** Completion rate (%) for each of the four writing sessions in the future work self writing and active control conditions. **(B)** Mean word count per writing session in the same two conditions; error bars denote 95% confidence intervals. **(C)** Future work self salience (1–5) at baseline (T1) and post-intervention (T2) in the three conditions; error bars denote 95% confidence intervals and the bracket marks the T1–T2 change in the future work self writing condition (^***^p < .001). The waitlist condition completed no writing sessions and therefore appears in panel **(C)** only.

### 3.5 Descriptive statistics and correlations

Table 7 reports means, standard deviations and correlations among all study variables at baseline.

**Table 7:** Means, standard deviations and correlations among study variables at baseline (*N* = 486).

Variable	M	SD	1	2	3	4	5	6
1. FWS salience	3.12	0.78						
2. Self-exploration	2.87	0.71	0.34**					
3. Env. exploration	2.66	0.76	0.29**	0.51**				
4. Rum. exploration	3.23	0.82	−0.31**	0.12*	0.08			
5. Lack of readiness	4.82	1.34	−0.28**	−0.06	−0.09	0.47**		
6. Lack of info.	5.15	1.41	−0.24**	−0.19**	−0.22**	0.33**	0.52**	
7. Inconsist. info.	4.92	1.31	−0.26**	−0.08	−0.11*	0.44**	0.58**	0.61**

^*^p < 0.05. ^**^p < 0.01.

### 3.6 Intervention effects on ruminative and reflective career exploration

Table 8 reports the latent growth curve results. Figure 5 displays the estimated trajectories by condition. Relative to both control conditions the future work self writing condition showed a significant decrease in ruminative career exploration [*d* = 0.41, 95% CI [0.22, 0.61], *p* < 0.001 vs. waitlist; *d* = 0.35, 95% CI [0.16, 0.55], *p* < 0.001 vs. active control]. Change in self-exploration was small and non-significant [*d* = 0.10, 95% CI [−0.10, 0.30], *p* = 0.31 vs. waitlist]. Change in environmental exploration was likewise non-significant [*d* = 0.13, 95% CI [−0.05, 0.32], *p* = 0.14 vs. waitlist]. These results support H2.

**Figure 5:**
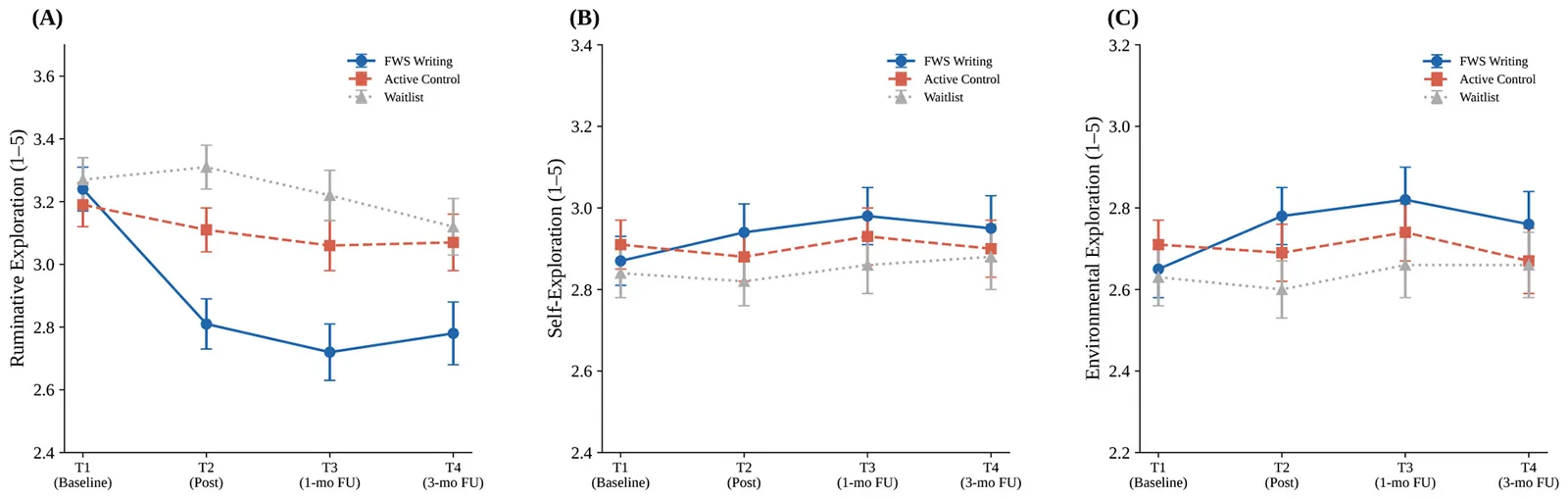
Estimated trajectories of career exploration across the four assessment waves by condition. **(A)** Ruminative career exploration. **(B)** Self-exploration. **(C)** Environmental exploration. Each panel plots the model-implied means for the future work self writing, active control and waitlist conditions at T1 (baseline), T2 (post-intervention), T3 (one-month follow-up) and T4 (three-month follow-up); error bars denote 95% confidence intervals. All three outcomes are measured on a 1–5 scale, but vertical axis is scaled separately in each panel.

**Table 8:** Latent growth curve model results for intervention effects on primary outcomes, with confidence intervals for both the unstandardized and the standardized effect.

Outcome and contrast	*b*	SE	95% CI for *b*	*p*	*d*	95% CI for *d*
Ruminative career exploration						
FWS vs. waitlist	−0.34	0.08	[−0.50, −0.18]	< 0.001	0.41	[0.22, 0.61]
FWS vs. active control	−0.29	0.08	[−0.45, −0.13]	< 0.001	0.35	[0.16, 0.55]
Self-exploration						
FWS vs. waitlist	0.07	0.07	[−0.07, 0.21]	0.31	0.10	[−0.10, 0.30]
FWS vs. active control	0.05	0.07	[−0.09, 0.19]	0.47	0.07	[−0.13, 0.27]
Environmental exploration						
FWS vs. waitlist	0.10	0.07	[−0.04, 0.24]	0.14	0.13	[−0.05, 0.32]
FWS vs. active control	0.09	0.07	[−0.05, 0.23]	0.19	0.12	[−0.07, 0.30]
Lack of readiness						
FWS vs. waitlist	−0.58	0.13	[−0.83, −0.33]	< 0.001	0.43	[0.25, 0.62]
FWS vs. active control	−0.51	0.13	[−0.76, −0.26]	< 0.001	0.38	[0.19, 0.57]
Lack of information						
FWS vs. waitlist	−0.16	0.14	[−0.43, 0.11]	0.24	0.11	[−0.08, 0.30]
FWS vs. active control	−0.12	0.14	[−0.39, 0.15]	0.38	0.09	[−0.11, 0.28]
Inconsistent information						
FWS vs. waitlist	−0.46	0.13	[−0.71, −0.21]	0.001	0.35	[0.16, 0.54]
FWS vs. active control	−0.39	0.13	[−0.64, −0.14]	0.002	0.30	[0.11, 0.49]

*b* is the model-implied between-condition difference at the final assessment (T4), obtained by multiplying the difference in the per-month slope factor by the T4 time loading of 4, and is expressed in the original metric of the outcome. *d* = *b* divided by the pooled baseline standard deviation of the outcome. The interval for *d* is obtained by applying the same transformation to the limits of the interval for *b*. *d* is reported as a positive quantity throughout; for the difficulty and rumination outcomes a positive *d* denotes a reduction in the future work self condition relative to the comparison condition.

Because a non-significant test does not by itself establish the absence of an effect, the two reflective exploration outcomes were also submitted to equivalence tests against a bound of *d* = 0.30, as specified in Section 2.7. Results are reported in Table 9. For every contrast the 90 percent confidence interval of the standardized effect fell entirely inside the equivalence bounds and the two one-sided tests were significant, with p values between 0.009 and 0.031. Effects on reflective career exploration of *d* = 0.30 or larger can therefore be rejected. Smaller effects cannot be rejected; against a bound of *d* = 0.20 the equivalence tests were not significant. The appropriate conclusion is that the intervention produced no detectable increase in reflective career exploration and no increase large enough for this trial to detect, rather than that the effect is exactly zero.

**Table 9:** Equivalence tests for the reflective career exploration outcomes.

Outcome and contrast	*d*	SE_*d*_	90% CI	TOST *p*
Self-exploration, FWS vs. waitlist	0.10	0.10	[−0.07, 0.27]	0.023
Self-exploration, FWS vs. active control	0.07	0.10	[−0.09, 0.23]	0.009
Environmental exploration, FWS vs. waitlist	0.13	0.09	[−0.02, 0.28]	0.031
Environmental exploration, FWS vs. active control	0.12	0.09	[−0.03, 0.27]	0.027

All four tests are significant at α = 0.05, supporting equivalence within ±0.30. The 90 percent interval is the interval appropriate to a two one-sided tests procedure at α = 0.05; the corresponding 95 percent intervals are reported in Table 8.

Two one-sided tests against a smallest effect size of interest of *d* = 0.30, computed from the latent growth model estimates in Table 8.

### 3.7 Differential effects across dimensions of career decision-making difficulties

Figure 6 displays effects across the three dimensions of the taxonomy. Relative to the waitlist condition, effects were largest on the lack of readiness dimension [*d* = 0.43, 95% CI [0.25, 0.62], *p* < 0.001]. Effects on inconsistent information were moderate [*d* = 0.35, 95% CI [0.16, 0.54], *p* = 0.001]. Effects on lack of information were small and non-significant [*d* = 0.11, 95% CI [−0.08, 0.30], *p* = 0.24]. The effect sizes quoted in this paragraph are the comparisons with the waitlist condition. Each panel of Figure 6 also annotates the comparison with the active control condition, namely 0.38, 0.09, and 0.30. That set is the more conservative of the two. Both sets are reported in Table 8.

**Figure 6:**
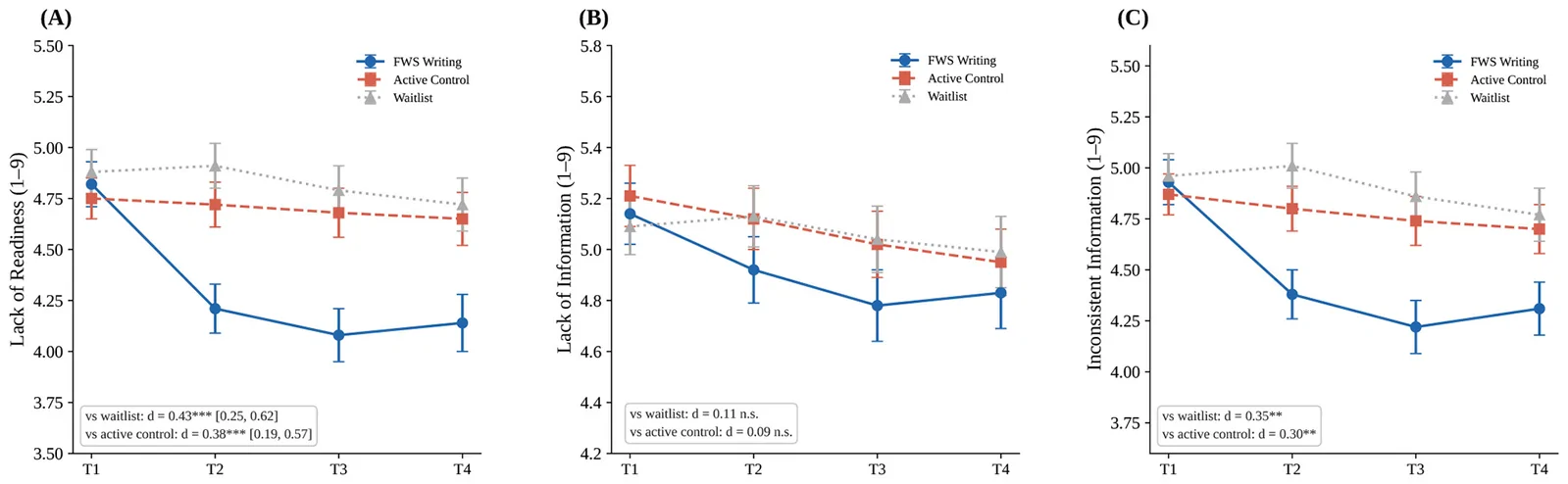
Differential intervention effects across the three dimensions of career decision-making difficulties by condition. **(A)** Lack of readiness. **(B)** Lack of information. **(C)** Inconsistent information. Each panel plots the model-implied means for the future work self writing, active control and waitlist conditions at T1 (baseline), T2 (post-intervention), T3 (one-month follow-up) and T4 (three-month follow-up) on the 1–9 scale of the taxonomy; error bars denote 95% confidence intervals and the vertical axis is scaled separately in each panel. The inset box in each panel annotates the contrast with the waitlist condition and the contrast with the active control condition, where ^**^p < 0.01, ^***^p < 0.001 and n.s. denotes a non-significant contrast. Confidence intervals for all six contrasts are reported in Table 8.

We state what this pattern does and does not establish, because the wording of the original submission was imprecise on the point. The three dimensions were modeled in separate growth models and the three contrasts were not compared with one another in a formal test, so the statement that the effect differs across dimensions is a description of three separately estimated quantities rather than the result of a test of their difference. What the analysis supports is the more specific claim made by H3 as stated in Section 1.5, namely that a significant reduction is obtained on lack of readiness and on inconsistent information and that no detectable reduction is obtained on lack of information. The confidence intervals reported above show the same asymmetry: the intervals for lack of readiness and inconsistent information exclude zero, whereas the interval for lack of information includes zero and includes values close to it. H3 is therefore reported as supported in that specific sense, and the pattern is consistent with the subtractive account and inconsistent with the additive account, which predicts the largest effect on the dimension where none was detected.

These are slope contrasts estimated across all four waves rather than tests of the difference between two specific waves. The statement that the effects persist to the 3-month follow-up therefore rests on the model-implied trajectories displayed in Figures 5, 6, in which the separation between the future work self condition and both control conditions is present at T2 and remains at T3 and T4, and on the fact that the slope contrasts remain significant when the observation period is extended to T4. We did not conduct a formal test of the change between T2 and T4, so we describe the effects as present at the 3-month follow-up rather than as formally shown to be maintained. The distinction is treated as a limitation in Section 4.4.

### 3.8 Mediation analysis and contrast of indirect effects

Table 10 reports the parallel multiple mediator model, estimated with the active control condition as the reference category. Figure 7 displays the model. All path coefficients shown in Figure 7 and all indirect effects in Table 10 are unstandardized and are estimated within this single mediation model; they are not imported from the latent growth curve models in Table 8. The *a*-paths represent the effect of condition assignment on the latent change score of each mediator from baseline to post-intervention, and because the mediation model uses a different parameterisation from the four-wave growth models, these coefficients need not equal the slope contrasts in Table 8. The product of the two paths in the figure reproduces the corresponding indirect effect in the table. The indirect effect through ruminative career exploration was −0.18 with a 95 percent bias-corrected confidence interval of [−0.29, −0.09]. The indirect effect through self-exploration was −0.02 with a 95 percent bias-corrected confidence interval of [−0.11, 0.06]. The indirect effect through environmental exploration was −0.03 with a 95 percent bias-corrected confidence interval of [−0.10, 0.05]. The formal contrast of the ruminative vs. self-exploration indirect effects was −0.16 with a 95 percent confidence interval of [−0.28, −0.05]. The formal contrast of the ruminative vs. environmental exploration indirect effects was −0.15 with a 95 percent confidence interval of [−0.27, −0.04]. The ruminative path accounted for approximately 35% of the total intervention effect on lack of readiness (−0.18 of −0.51) and for approximately 78% of the combined indirect effect transmitted through the three mediators (−0.18 of −0.23), with a remaining direct effect of −0.28. These results support H4.

**Figure 7:**
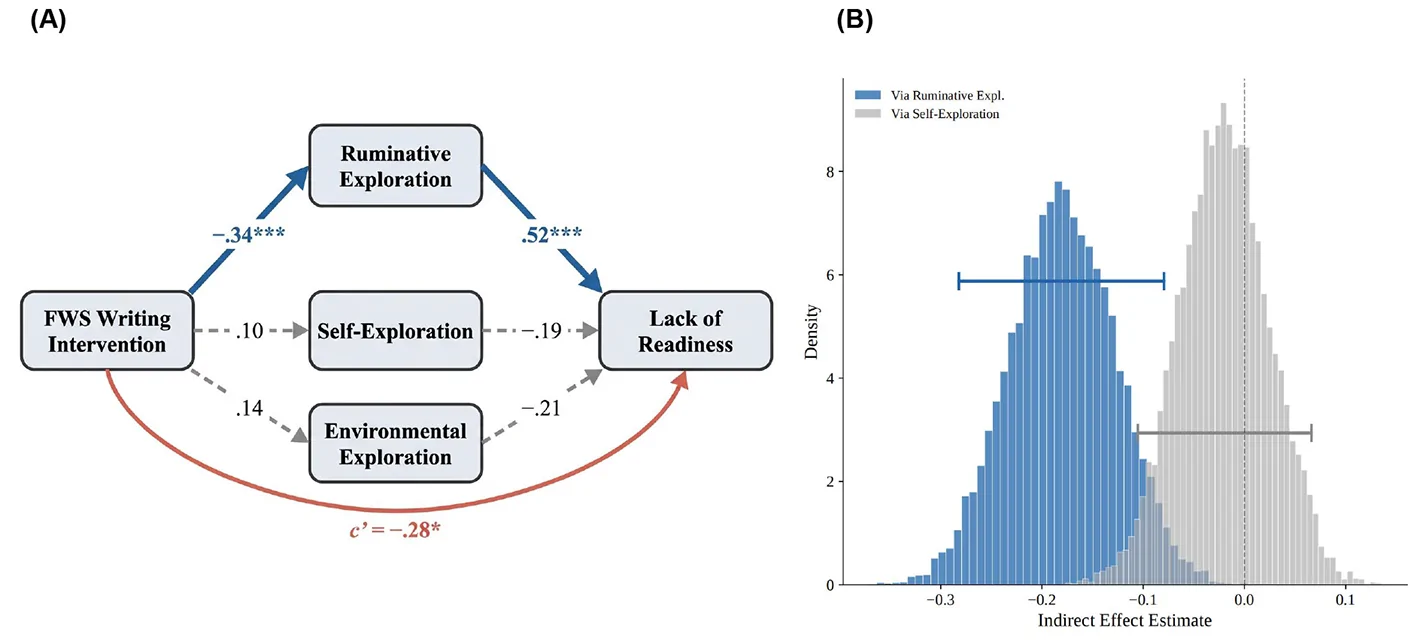
Parallel multiple mediator model with unstandardised path coefficients and bootstrap distributions of indirect effects with their formal contrast. **(A)** Path diagram of the model, in which the future work self writing intervention predicts lack of readiness at the one-month follow-up through change in ruminative career exploration, self-exploration and environmental exploration. Solid arrows denote significant paths and dashed arrows non-significant paths; c' is the direct effect of condition on lack of readiness with all three mediators in the model. **(B)** Bias-corrected bootstrap distributions of the indirect effect through ruminative career exploration and of the indirect effect through self-exploration, with the horizontal bars giving the corresponding 95% confidence intervals and the vertical dashed line marking zero. ^*^p < 0.05, ^***^p < 0.001. All coefficients are estimated within the mediation model itself and are not drawn from the latent growth curve models in Table 8. The a-paths represent the effect of condition on the latent change score of each mediator from baseline to post-intervention; the b-paths represent the association between mediator change and the level of lack of readiness at the one-month follow-up, controlling for baseline. Because the two model families use different parameterisations, numerical correspondence between the a-paths here and the slope contrasts in Table 8 is a feature of the data rather than of the specification.

**Table 10:** Parallel multiple mediator model with bias-corrected bootstrap confidence intervals and formal contrast of indirect effects (unstandardized estimates).

Effect	Est.	SE	CI lower	CI upper
Total on lack of readiness	−0.51	0.13	−0.76	−0.26
Indirect via ruminative expl.	−0.18	0.05	−0.29	−0.09
Indirect via self-exploration	−0.02	0.04	−0.11	0.06
Indirect via env. exploration	−0.03	0.04	−0.10	0.05
Direct effect	−0.28	0.12	−0.52	−0.04
Contrast rum. vs. self-expl.	−0.16	0.06	−0.28	−0.05
Contrast rum. vs. env. expl.	−0.15	0.06	−0.27	−0.04
Total on inconsist. info.	−0.39	0.13	−0.64	−0.14
Indirect via ruminative expl.	−0.14	0.05	−0.25	−0.06
Indirect via self-exploration	−0.01	0.03	−0.08	0.05
Indirect via env. exploration	−0.02	0.03	−0.09	0.04
Direct effect	−0.22	0.12	−0.46	0.02

These quantities describe the decomposition of the total effect within the fitted model. They should not be read as showing that the intervention reduced difficulties because it reduced rumination. The mediator was measured rather than manipulated, and the estimates rest on the untestable assumption that no unmeasured variable confounds the relationship between the change in ruminative career exploration and the subsequent level of lack of readiness (Imai et al., 2010). The substantial direct effect that remains, which is larger than the mediated portion, indicates in addition that most of the intervention effect is not accounted for by any of the three measured mediators. We therefore report H4 as supported in the sense that the pattern of indirect effects is consistent with the subtractive account and inconsistent with the additive account, without asserting that the causal mechanism has been established.

Two further cautions attach to the figure of 35 percent specifically. It is a ratio of two estimated quantities and inherits the uncertainty of both, so it is considerably less stable than either the total effect or the indirect effect from which it is formed, and no confidence interval is reported for it because the ratio is not the quantity the model was fitted to estimate. It is also not the larger of the two proportions that can be computed from the same model: the same indirect effect corresponds to 78 percent of the combined indirect effect through the three mediators, and we report both denominators so that the smaller and more conservative figure cannot be read in isolation. The interpretable quantities are the indirect effect itself, −0.18 with a bias-corrected interval of [−0.29, −0.09], and its formal contrasts with the two reflective pathways.

### 3.9 Employment outcomes at 3-month follow-up

Table 11 reports the four employment indicators in full, with descriptive values by condition, the test statistic, the effect estimate with its confidence interval and both the uncorrected and the Bonferroni-corrected p value. The values are given in the text and in the table rather than only in Figure 8. Conditions did not differ significantly on signed agreement status [χ^2^(2) = 3.21, *p* = 0.20] or on number of offers [*F*(2, 412) = 1.87, *p* = 0.16]. The future work self condition reported marginally higher satisfaction with employment quality [*d* = 0.19, 95% CI [−0.01, 0.40], *p* = 0.06] and nominally higher perceived field match [*d* = 0.24, 95% CI [0.03, 0.45], *p* = 0.03]. All four tests are unadjusted between-condition comparisons, as are the condition values reported in Table 11; because these indicators are exploratory and the conditions did not differ at baseline (Section 3.2), no covariate adjustment was applied. These four indicators form a single family of exploratory tests. After Bonferroni correction within that family the adjusted p values are 0.80 for signed agreement status, .64 for number of offers, 0.24 for satisfaction with employment quality and 0.12 for perceived field match. No employment outcome is significant after correction. The uncorrected difference in perceived field match corresponds to a small effect and the confidence interval for that estimate includes values close to zero. We therefore report that the trial found no evidence that the intervention improved employment outcomes at the 3-month follow-up, and treat the difference in perceived field match as a hypothesis-generating observation that requires replication in a trial powered for that endpoint. These results provide no more than weak and provisional support for the exploratory H5.

**Figure 8:**
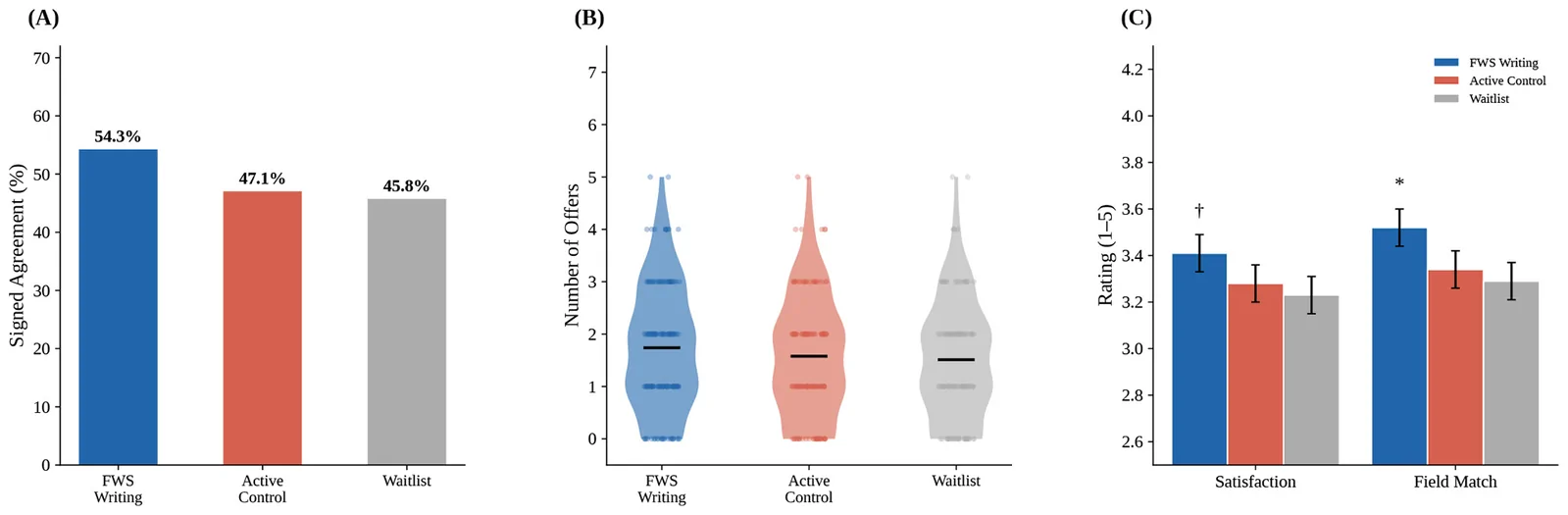
Employment outcomes at three-month follow-up by condition. **(A)** Percentage of participants who had signed an employment agreement, with the percentage printed above each bar. **(B)** Number of offers received, shown as violin plots with the individual observations overlaid and the horizontal line marking the condition mean. **(C)** Satisfaction with the employment outcome and perceived match between the position and the field of study, both rated on a 1–5 scale; error bars denote 95% confidence intervals and the markers above the future work self writing bars refer to the contrast with the waitlist condition (†*p* < 0.10, ^*^p < .05). Significance markers refer to uncorrected tests; no employment outcome remains significant after correction for the four comparisons. Numerical values and confidence intervals are reported in Table 11.

**Table 11:** Employment indicators at the 3-month follow-up by condition.

Indicator	FWS	Active ctrl	Waitlist	Test	Effect [95% CI]	*p*	*p*_adj_
Signed agreement (%)	54.3	47.1	45.8	χ^2^(2) = 3.21	OR 1.41 [0.87, 2.28]	0.20	0.80
Number of offers	1.74 (1.21)	1.58 (1.18)	1.51 (1.15)	*F*(2, 412) = 1.87	*d* 0.17 [−0.04, 0.38]	0.16	0.64
Satisfaction	3.41 (0.94)	3.28 (0.91)	3.23 (0.89)	*F*(2, 412) = 2.84	*d* 0.19 [−0.01, 0.40]	0.06	0.24
Field match	3.52 (0.97)	3.34 (0.93)	3.29 (0.91)	*F*(2, 412) = 3.51	*d* 0.24 [0.03, 0.45]	0.03	0.12

Values reported for each condition are unadjusted descriptive statistics: percentages for signed employment agreement status and *M* (SD) for the remaining three indicators. The test statistics and the effect estimates are unadjusted between-condition comparisons; no covariate adjustment was applied to these exploratory indicators. Effect estimates for number of offers, satisfaction and field match are the contrasts of the future work self condition with the waitlist condition. *p*_adj_ is the Bonferroni-corrected value within the family of four indicators; that correction was applied in response to peer review and was not part of the original analysis plan. No indicator is significant after correction.

All four indicators are single-item self-reports and form one family of exploratory tests; *p*-values are reported both uncorrected and after Bonferroni correction within that family.

### 3.10 Sensitivity analyses

Per-protocol and complier average causal effect estimates are reported in Supplementary Table S3. The per-protocol estimates were slightly larger than the intention-to-treat estimates. Results were robust to Bonferroni correction for multiple comparisons across the three difficulty dimensions. Within that family the adjusted p value remains below 0.003 for lack of readiness and is 0.003 for inconsistent information in the comparison with the waitlist condition, while lack of information remains non-significant. The two reflective exploration outcomes are non-significant with and without correction. The four employment indicators are corrected as reported in Section 3.9; that correction was applied in response to peer review and was not part of the original analysis plan. The primary conclusions were unchanged.

## 4 Discussion

### 4.1 Summary of findings

This trial tested two competing mechanisms for the effect of a future work self writing intervention on career decision-making difficulties. The results support the subtractive mechanism. The intervention reduced ruminative career exploration and this reduction statistically accounted for part of the effect on career decision-making difficulties. The intervention produced no detectable increase in reflective career exploration, and effects on that outcome as large as *d* = 0.30 could be rejected by equivalence testing. Effects were concentrated on lack of readiness and inconsistent information rather than on lack of information. This pattern aligns with the prediction that clarifying a hoped-for work identity interrupts unresolved repetitive thought rather than stimulating additional information search. Three qualifications frame everything that follows. The comparison of conditions is randomized and supports causal inference about the intervention as a whole, whereas the decomposition into indirect and direct effects is model-based and rests on assumptions that the design cannot verify. The effects that persist to the 3-month follow-up are described from the estimated trajectories over four waves rather than from a formal test of change between the post-intervention assessment and the final assessment. And the differential pattern across the three difficulty dimensions is a description of three separately estimated contrasts rather than the result of a formal comparison between them.

### 4.2 Theoretical implications

The first implication concerns the exploration paradox. The heterogeneity documented by Jiang et al. (2019) and subsequently confirmed by Kleine et al. (2021) has usually been attributed to unmeasured moderators. Our results support an alternative account in which the exploration construct is itself heterogeneous. If exploration comprises a reflective and a ruminative form then studies that measure only aggregate exploration will produce unstable estimates. The instability is a measurement artifact rather than a substantive contingency. The specific result that carries this claim is the contrast between the two kinds of exploration within the same trial and the same sample: the ruminative form moved reliably [*d* = 0.41, 95% CI [0.22, 0.61] vs. waitlist] while the two reflective subscales did not move detectably and were statistically equivalent to zero within a bound of *d* = 0.30. A measure that aggregated the two would have registered the average of a moving component and two static components, which is exactly the situation in which pooled estimates become unstable across samples. Lee et al. (2023) showed that self-exploration and environmental exploration relate differently to personality traits. Our findings add a further layer of heterogeneity within the career exploration construct itself.

The second implication concerns the mechanism of future work self interventions. Prior work has framed the future work self as a motivational amplifier that generates additional proactive behavior (Strauss et al., 2012; Parker et al., 2010; Taber and Blankemeyer, 2015). The recent systematic review by Talluri et al. (2025) noted that the field has predominantly examined behavioral and developmental outcomes and called for research on emotional and cognitive outcomes. Our findings are consistent with a subtractive account in which the intervention operates by interrupting unresolved repetitive thought. Under the subtractive account a salient future work self functions as an organizing standard rather than as an energiser. This reading is supported by two independent features of the results, namely the absence of any detectable increase in reflective exploration and the concentration of effects on the readiness dimension [*d* = 0.43, 95% CI [0.25, 0.62]] and the consistency dimension [*d* = 0.35, 95% CI [0.16, 0.54]] rather than on the information dimension [*d* = 0.11, 95% CI [−0.08, 0.30]]. It remains an interpretation of a pattern of effects rather than a demonstrated causal chain.

The role of future work self salience itself deserves separate comment, since it is the construct the intervention was designed to move and the trial provides direct evidence about it. The manipulation check confirmed that the writing task did what it was designed to do, producing a larger increase in salience from baseline to post-intervention than either the active control or the waitlist condition. Because the active control was matched on session duration, frequency, response format and expected word count and did not produce the same increase, the downstream effects cannot be attributed to structured writing in general. Change in salience was not entered as a mediator, and the reason is a matter of design rather than of oversight: the mediation model specified by H4 contrasts the two exploration pathways, which are the quantities on which the additive and the subtractive accounts disagree, whereas salience is the construct the manipulation targets, and using it simultaneously as the manipulation check and as a mediator would confound the test of the two rival pathways. The serial model in which salience mediates the effect on rumination, which in turn mediates the effect on readiness, is the natural extension of the present design; it was not specified in the analysis plan and is proposed in Section 4.5 rather than estimated here. The pattern is nonetheless informative for theory. Salience increased while reflective exploration did not, so in this sample salience is not behaving as the motivational amplifier that proactive motivation theory describes (Parker et al., 2010), and the dissociation between the two is itself evidence for reading its function as organizing rather than energizing.

The third implication concerns the transfer of identity development constructs into vocational psychology. The ruminative exploration dimension of Luyckx et al. (2008) was developed for the domain of general future plans. Our results support its applicability to the occupational domain. The discriminant validity evidence reported in Table 3 establishes boundaries against general indecisiveness (Gati et al., 1996), trait self-rumination (Trapnell and Campbell, 1999) and career reconsideration (Porfeli et al., 2011). That evidence is bounded in two ways, in that it was obtained in the same sample as the substantive findings and that no convergent coefficient against an established measure of distress is available, so the psychometric case for the adapted scale should be regarded as promising rather than settled. The cross-cultural validation evidence from Rivnyák et al. (2022) and Sánchez-Queija et al. (2024) further supports the robustness of this distinction across populations.

The fourth implication concerns the differential effects across dimensions of career decision-making difficulties. The finding that the intervention primarily reduced lack of readiness and inconsistent information is consistent with the dual-process theory of career decision-making (Xu, 2023a). That framework highlights the role of ambiguity management processes in career decision-making. Our intervention may have facilitated ambiguity management by providing a coherent future identity that reduced the perceived inconsistency among available career options. This last statement is an interpretation rather than a finding, since ambiguity management was not measured in this trial.

### 4.3 Practical implications for career guidance

University career services in China typically operate on the assumption that students should explore more. Career fairs, internship databases and information sessions all increase the volume of exploration opportunities. The recent trend toward online career interventions for Chinese students (Chen et al., 2022) follows a similar logic. Our findings suggest that volume is the wrong target for a substantial subgroup of students.

Three concrete recommendations follow. Each is a suggestion for practice that follows from a single trial and should be adopted provisionally. First, career services can screen for ruminative career exploration during the first term of the final year. Students high on this dimension are unlikely to benefit from additional information provision. The adapted scale used here is not yet validated to a standard that would justify using it to allocate individual students to services, and any such use should await independent validation. Second, a four-session structured writing module can be embedded into existing career courses. We state the resource requirement rather than describing the cost as negligible, since no economic evaluation was conducted. In this trial the module consisted of four sessions of approximately 20 min, giving a total student time commitment of about 80 min across 4 weeks; it was delivered through an existing online platform with an automated reminder protocol, required no individual contact time and no specialist counseling staff, and the only recurring input was the fidelity coding, which was carried out for research purposes and would not be required in routine delivery. The marginal cost of each additional student is therefore limited to platform capacity. This is a statement about delivery resources and not a claim of cost-effectiveness, which would require the economic evaluation that this trial did not perform. Third, guidance content can shift from how much students explore toward how students explore. Practitioners can teach concrete and problem-focused processing rather than abstract evaluative processing (Nolen-Hoeksema et al., 2008). We note that the trial found no evidence of effects on employment outcomes, so these recommendations are supported for psychological outcomes during the transition and not for labor market results. They are also supported for the setting in which the trial was conducted rather than for university career services generally, for the reasons set out in the second limitation below.

### 4.4 Limitations

Twelve limitations warrant attention. First, all psychological outcomes relied on self-report. Behavioral indicators of exploration were not collected.

Second, and in our judgement the most consequential boundary on the findings, the trial was conducted in a single national context whose graduate labor market has several features that the intervention may depend on, and generalization beyond that context is an open empirical question rather than a matter of degree. The Chinese graduate recruitment calendar is compressed and institutionally driven, with an autumn season running from September to November, a spring season from March to April and graduation in June; that calendar concentrates career-related thought into a defined window and may therefore raise both the baseline level of ruminative career exploration and the scope for a brief intervention to interrupt it. Where hiring is continuous, career-related repetitive thought may be more diffuse and the same four sessions may have less to interrupt. The cohort is also unusually large, at 11.79 million graduates in 2024 (Wang and Wang, 2024), with competitive pressure to match, and institutional career guidance is organized around mandatory credit-bearing courses, which is the delivery channel that the recommendations in Section 4.3 assume. Within China, the four participating universities were drawn from the eastern region and span two institutional tiers; that design addresses the convenience-sample problem at the level of the site but does not make the sample representative of Chinese universities regionally or nationally, and eastern institutions face labor market conditions that differ from those in central and western provinces. Cross-cultural replication is accordingly listed in Section 4.5 as a substantive test of the mechanism rather than as a routine extension.

Third, participants could not be blinded to condition. Expectancy effects cannot be fully excluded despite the active control. Fourth, the intervention was brief and delivered online. Longer or facilitated formats might produce different effects. Fifth, the 3-month follow-up may be too short to capture durable change. Sixth, employment outcomes are sensitive to macroeconomic conditions beyond the control of any individual intervention.

Seventh, the central mediator was measured with a scale that was adapted for this trial. Its confirmatory factor structure and its discriminant validity against general indecisiveness, trait self-rumination and career reconsideration were established in the trial baseline sample rather than in an independent sample, so those indices are not cross-validated and are likely to be optimistic. No established measure of distress or negative affect was administered, so the definitional claim that ruminative career exploration generates distress is supported by the theoretical literature and by the pattern of correlations reported in Table 7 rather than by a direct convergent coefficient. The scale should be treated as provisional until it is validated in an independent sample against external criteria, and every conclusion that depends on it inherits that provisional status.

Eighth, clustering of participants within the four participating universities was not modeled with a random intercept, for the reasons set out in Section 2.7. Randomization was stratified by university, so the treatment contrasts are not confounded with institution, but standard errors may be somewhat too small if within-university similarity is substantial, and the precision of the estimates is correspondingly overstated to an unknown degree.

Ninth, the mediation analysis establishes temporal ordering but not causal identification. The mediator was measured rather than manipulated, sequential ignorability cannot be tested with these data, and no sensitivity analysis for unmeasured mediator-outcome confounding was conducted (Imai et al., 2010). The direct effect remains larger than the mediated portion, so most of the intervention effect is not explained by any measured mediator.

Tenth, the number of statistical tests reported is large relative to the number of participants. Corrections were applied within families of outcomes, and the correction within the employment family was added after peer review rather than pre-specified. The two primary outcomes survive correction comfortably, but the exploratory findings, and the employment findings in particular, should be regarded as provisional.

Eleventh, the growth models specify a linear slope, whereas the estimated trajectories indicate a step-like change between baseline and the post-intervention assessment followed by a plateau. The linear specification therefore summarizes the shape of change rather than reproducing it, and it is the most likely source of the residual misfit reported in Section 3.3. It also constrains what the analysis can say about persistence, because separating the immediate change from the subsequent period requires a parameterization that this trial did not specify. Reanalysis with a piecewise growth model or with freely estimated slope loadings is a priority for any replication.

Twelfth, the treatment of missing data rests on an assumption that cannot be verified. Full information maximum likelihood requires data to be missing at random, and although retention was high and non-differential and no baseline variable predicted dropout, neither of these observations rules out a mechanism in which the reason for dropping out is related to the unobserved outcome value itself (Graham, 2009). No analysis under an explicit departure from that assumption was conducted, and the estimates should be read as conditional on it.

### 4.5 Future directions

Four directions follow from the present work. Ecological momentary assessment could establish whether ruminative career exploration fluctuates within persons across the recruitment season. Dismantling designs could isolate which of the four writing sessions carries the effect. Cross-cultural replication could test whether the subtractive mechanism holds where hiring is continuous rather than seasonal. Integration with the career self-management framework (Lent and Brown, 2020) could extend the model beyond the school-to-work transition by examining how the interplay between future work selves and rumination operates across later career transitions.

A dedicated validation study of the adapted ruminative career exploration scale is a precondition for several of these directions. Such a study should recruit an independent sample, administer an established measure of distress or negative affect alongside the career-domain items so that a convergent coefficient can be estimated directly, cross-validate the factor structure, and examine measurement invariance across institutional and disciplinary groups. Randomizing the mediator itself, for example by comparing a writing task designed to interrupt repetitive thought with one designed to increase information search, would provide the experimental test of the mechanism that the present design cannot supply. A further extension would estimate the serial model discussed in Section 4.2, in which change in future work self salience predicts change in ruminative career exploration, which in turn predicts the subsequent level of lack of readiness; that model requires an assessment schedule dense enough to separate the three changes in time, which the present four-wave design does not provide.

## 5 Conclusion

This three-arm randomized controlled trial found that a four-session future work self writing intervention reduced ruminative career exploration and reduced the lack of readiness and inconsistent information dimensions of career decision-making difficulties, relative both to an active control and to a waitlist control. No detectable increase in reflective career exploration was obtained, and increases as large as *d* = 0.30 could be rejected by equivalence testing. Change in ruminative career exploration was associated with the reduction in lack of readiness, whereas change in reflective career exploration was not, and the two indirect effects differed from each other; these are model-based associations rather than a demonstrated causal chain, and most of the total effect was not transmitted through any measured mediator. No employment indicator remained significant after correction within its family, so the trial provides no evidence of labor market benefit at 3 months. The evidence is therefore consistent with the interpretation that the benefit of clarifying a future work self lies in interrupting unproductive career rumination rather than in stimulating additional exploration, and it suggests that career guidance may repay attention to how students explore and not only to how much, in settings resembling the one studied here.

## Data Availability

The original contributions presented in the study are included in the article/Supplementary material, further inquiries can be directed to the corresponding author.
