# Supplementary material for: Effects of a future work self writing intervention on ruminative career exploration and career decision-making difficulties among final-year university students: a three-arm randomized controlled trial
Source: Front Psychol. 2026 Oct 7;17:1965900. doi: 10.3389/fpsyg.2026.1965900 (PMC13683202; doi:10.3389/fpsyg.2026.1965900)
Supplement: Supplementary file 1 [file Table_1.docx]

**Supplementary Material**

**Supplementary Table S1. CONSORT 2010 Checklist**

| **Item** | **Topic** | **Checklist item** | **Reported on** |
| --- | --- | --- | --- |
| 1a | Title | Identification as a randomised trial in the title | Title page |
| 1b | Title | Structured summary of trial design, methods, results, and conclusions | Abstract |
| 2a | Background | Scientific background and explanation of rationale | Section 1 |
| 2b | Objectives | Specific objectives or hypotheses | Section 1.5 |
| 3a | Trial design | Description of trial design including allocation ratio | Section 2.1 |
| 3b | Trial design | Important changes to methods after trial commencement with reasons | Section 2.1 |
| 4a | Participants | Eligibility criteria for participants | Section 2.2 |
| 4b | Participants | Settings and locations where the data were collected | Section 2.2; Table S6 |
| 5 | Interventions | Interventions for each group with sufficient detail | Section 2.4 |
| 6a | Outcomes | Completely defined pre-specified primary and secondary outcome measures | Sections 2.5, 2.5.7 |
| 6b | Outcomes | Any changes to trial outcomes after the trial commenced with reasons | Section 2.1 |
| 7a | Sample size | How sample size was determined | Section 2.2 |
| 7b | Sample size | Explanation of any interim analyses and stopping guidelines | N/A |
| 8a | Randomisation sequence | Method used to generate the random allocation sequence | Section 2.3 |
| 8b | Randomisation sequence | Type of randomisation and details of restriction | Section 2.3 |
| 9 | Allocation concealment | Mechanism used to implement the random allocation sequence | Section 2.3 |
| 10 | Implementation | Who generated the allocation sequence, enrolled and assigned participants | Section 2.3 (three roles now reported separately) |
| 11a | Blinding | Who was blinded after assignment to interventions and how | Section 2.3 |
| 11b | Blinding | Description of the similarity of interventions | Section 2.4.2 |
| 12a | Statistical methods | Statistical methods used to compare groups for primary and secondary outcomes | Section 2.7 |
| 12b | Statistical methods | Methods for additional analyses such as subgroup analyses and adjusted analyses | Section 2.7; missing-data assumptions and attrition analysis in Section 2.7 and Table S7 |
| 13a | Participant flow | Numbers of participants randomly assigned, received intended treatment, analysed | Figure 3, Section 3.1 (available-case and intention-to-treat numbers distinguished) |
| 13b | Participant flow | Losses and exclusions after randomisation with reasons | Figure 3, Section 3.1 |
| 14a | Recruitment | Dates defining the periods of recruitment and follow-up | Sections 2.2, 2.6 |
| 14b | Recruitment | Why the trial ended or was stopped | Planned end reached |
| 15 | Baseline data | Baseline demographic and clinical characteristics of each group | Table 4 |
| 16 | Numbers analysed | Number of participants in each analysis and whether intention-to-treat | Section 3.1 |
| 17a | Outcomes | For each primary and secondary outcome, results for each group and estimated effect size | Tables 8-11, Figures 5-8 (all effect sizes with confidence intervals) |
| 17b | Outcomes | For binary outcomes, both absolute and relative effect sizes recommended | Section 3.9, Table 11 |
| 18 | Ancillary analyses | Results of any other analyses performed, including subgroup analyses and adjusted analyses, distinguishing pre-specified from exploratory | Sections 2.5.7 and 3.10; Table S3 |
| 19 | Harms | Important harms or unintended effects in each group | No harms reported |
| 20 | Limitations | Trial limitations, addressing sources of potential bias and imprecision | Section 4.4 (twelve limitations) |
| 21 | Generalisability | Generalisability of the trial findings | Section 4.4 |
| 22 | Interpretation | Interpretation consistent with results, balancing benefits and harms | Sections 4.1-4.3; Section 5 |
| 23 | Registration | Registration number and name of trial registry | Section 2.1 |
| 24 | Protocol | Where the full trial protocol can be accessed if available | Registry record ChiCTR2500074449 |
| 25 | Funding | Sources of funding and other support, role of funders | Funding section |

*Note. Item numbers follow the CONSORT 2010 checklist. The declaration of interests is not a CONSORT 2010 checklist item; it is reported in the Conflict of Interest Statement of the main manuscript.*

**Supplementary Table S2. Fidelity Coding Rubric**

Two independent raters coded a random sample of approximately 20% of the participants who received a writing condition (n = 65 of 319 participants), coding every submission made by each sampled participant using the rubric below. Each submission received a score of 0, 1, or 2 on each of five criteria. A submission was classified as on-topic if it received a score of 2 on at least three of the five criteria. Interrater agreement on the binary on-topic classification was κ = .91.

| **Criterion** | **Score 0** | **Score 1** | **Score 2** |
| --- | --- | --- | --- |
| On-topic content | No reference to work or career | Mentions work or career but no elaboration | Describes a future work self with concrete detail |
| Temporal orientation | Describes only the present | References the future but vaguely | Describes a specific future time point |
| Self-referential framing | Describes others or abstract situations | Uses first person but generically | Clearly describes the self in a future role |
| Elaboration depth | Fewer than 50 words | 50 to 150 words with limited detail | More than 150 words with specific detail |
| Emotional engagement | No affect words detected | One or two affect words present | Clear positive or negative affect expressed |

**Supplementary Table S3. Sensitivity Analyses**

All sensitivity analyses replicate the pattern reported in the primary intention-to-treat analysis. Per-protocol estimates include only participants who completed three or more of the four writing sessions. CACE estimates adjust for non-compliance using an instrumental variable approach. Bonferroni correction adjusts the critical alpha from .05 to .017 for the three CDDQ dimension tests.

| **Analysis** | **Rum. d** | **Rum. p** | **LoR d** | **LoR p** | **II d** | **II p** | **Conclusion** |
| --- | --- | --- | --- | --- | --- | --- | --- |
| ITT (primary) | 0.41 | <.001 | 0.43 | <.001 | 0.35 | .001 | Supported |
| Per-protocol (>=3 sessions) | 0.47 | <.001 | 0.49 | <.001 | 0.39 | <.001 | Supported |
| CACE estimation | 0.44 | <.001 | 0.46 | <.001 | 0.37 | .001 | Supported |
| Bonferroni-adjusted (α = .017) | 0.41 | <.001 | 0.43 | <.001 | 0.35 | .001 | Supported |

*Note. Rum. = ruminative career exploration (FWS vs waitlist); LoR = lack of readiness (FWS vs waitlist); II = inconsistent information (FWS vs waitlist). All d values are standardised mean differences from latent growth curve models. Lack of information, the third dimension in the career decision-making difficulty family, is not tabulated: the effect on that dimension is non-significant in the primary analysis (d = 0.11, 95% CI [-0.08, 0.30], p = .24; Table 8 of the main manuscript) and a correction that can only raise the adjusted p value cannot make it significant. The Bonferroni-adjusted critical alpha of .017 applies within that family of three dimensions; ruminative career exploration is a separate primary outcome and is not a member of it.*

Multiplicity. Corrections were applied within families of outcomes rather than across the whole set of tests. Within the family of the three career decision-making difficulty dimensions the Bonferroni-adjusted critical alpha is .017, as shown in the table above; equivalently, and as reported in Section 3.10 of the main manuscript, the adjusted p value remains below .003 for lack of readiness and is .003 for inconsistent information, while lack of information remains non-significant. The conclusions are unchanged. Within the family of the four employment indicators, which was not corrected in the original submission and was corrected in response to peer review, the adjusted p values are .80 for signed employment agreement status, .64 for number of offers received, .24 for satisfaction with employment quality and .12 for perceived field match; no employment outcome is significant after correction. Within the family of the two reflective career exploration subscales all tests are non-significant with and without correction, and equivalence tests against a smallest effect size of interest of d = 0.30 are reported in Table 9 of the main manuscript.

Descriptive values, effect estimates with 95% confidence intervals and both uncorrected and corrected p values for the four employment indicators are reported in Table 11 of the main manuscript.

**Supplementary Table S4. Full Item Pool for the Adapted Ruminative Career Exploration Scale**

Items were adapted from the ruminative exploration subscale of the Dimensions of Identity Development Scale (Luyckx et al., 2008). The original items reference general future plans and were rewritten to reference occupational content. Three vocational psychology researchers reviewed the adapted items for domain relevance. Translation and back-translation followed Brislin (1970). The item pool was administered to an independent pilot sample (n = 185). Item 8 was dropped because its factor loading (.73) fell below the a priori retention criterion of .75. The final 7-item scale demonstrated strong internal consistency (ω = .91 in the trial baseline sample).

Exploratory factor analysis procedure. The pilot sample of 185 respondents corresponds to a respondent-to-item ratio of approximately 23:1. Factorability of the correlation matrix was checked before extraction: the Kaiser-Meyer-Olkin measure of sampling adequacy was .91 and Bartlett's test of sphericity was significant, chi-square(28) = 987.64, p < .001. Factors were extracted by principal axis factoring and the number of factors to retain was determined by eigenvalues greater than one together with inspection of the scree plot. A single factor was retained and no rotation was applied. The extracted factor accounted for 62.3 percent of the item variance with an eigenvalue of 4.98; communalities ranged from .53 to .71 and corrected item-total correlations from .67 to .78 across the eight candidate items. The same information is reported in Section 2.5.3 and in the note to Table 2 of the main manuscript.

Expert item review. The three reviewers held doctoral degrees in vocational or organisational psychology and each had published peer-reviewed work on career development. They rated every candidate item independently on a four-point scale for relevance to the target construct and for clarity of wording, and were asked to flag any item that could be read as referring to general life planning, to a settled occupational commitment or to domain-general rumination rather than to the career exploration process itself. Items rated as not relevant or as ambiguous by any reviewer were rewritten and re-rated until agreement was reached. The reviewers took no part in data collection or analysis.

Boundaries of the present psychometric evidence. The confirmatory factor analysis and the discriminant validity comparisons reported in Table 3 of the main manuscript were estimated in the trial baseline sample rather than in an independent sample and are therefore not cross-validated, and no established measure of distress or negative affect was administered, so no direct convergent validity coefficient is available. The conceptual grounds on which ruminative career exploration is distinguished from domain-general rumination are set out in Section 1.4 of the main manuscript and the corresponding empirical separation is tested in Section 2.5.3. Both boundaries are stated in Section 2.5.3 and in the Limitations of the main manuscript. In the two-factor and merged models reported in Table 3 of the main manuscript each neighbouring construct was represented by parcelled indicators, which is why the degrees of freedom are smaller than the item counts in Table 1 of the main manuscript imply.

| **Item** | **English wording** | **Chinese wording** | **Status** |
| --- | --- | --- | --- |
| 1 | I keep asking myself whether my career plan is right | 我不断质疑自己的职业规划是否正确 | Retained |
| 2 | I worry repeatedly about what career suits me | 我反复担忧什么职业适合我 | Retained |
| 3 | I doubt my career choices without reaching a conclusion | 我对职业选择产生怀疑但无法得出结论 | Retained |
| 4 | I go over and over the same career concerns in my mind | 我脑海中反复回想同样的职业困惑 | Retained |
| 5 | I feel stuck cycling through the same occupational doubts | 我感到困在同样的职业疑虑中无法脱身 | Retained |
| 6 | I cannot stop thinking about career problems I cannot solve | 我无法停止思考那些我解决不了的职业问题 | Retained |
| 7 | I keep comparing career options without making progress | 我不断比较职业选择却毫无进展 | Retained |
| 8 | I revisit the same career worries day after day | 我每天都会重新回到同样的职业担忧 | Dropped (r_it = .67; loading = .73, below .75 retention criterion) |

**Supplementary Material S5. Complete Writing Instructions**

*Structured writing instructions apply to the two writing conditions only. Participants in the waitlist control condition completed the assessments without a writing task and received the full future work self writing programme after the three-month follow-up assessment.*

**S5.1 Future Work Self Writing Condition**

*Instructions were presented on the online platform at the beginning of each session. Participants were given 20 minutes to complete each writing task.*

**Session 1: Imagining Your Future Work Self**

Please take a moment to think about your working life several years from now. Imagine the best possible version of yourself at work. What kind of work are you doing? Where are you working? What does your workplace look like? What skills are you using every day? Write about this future work self in as much concrete detail as you can. Describe what you see, what you feel and what you do in a typical moment at work. There are no right or wrong answers. Write freely for about 20 minutes.

**Session 2: A Day in the Life of Your Future Work Self**

Think again about the future work self you described last week. Now imagine a typical working day for that person. What time do you start? Who do you interact with? What tasks fill your morning and your afternoon? How do you feel during the day? Describe this day from beginning to end in as much detail as you can.

**Session 3: Capabilities and Relationships**

Consider the future work self you have been writing about. What capabilities does that person possess? These might include professional skills, knowledge areas, personal qualities or habits. What professional relationships does that person maintain? Describe the capabilities and relationships that make your future work self effective and fulfilled.

**Session 4: Connecting Present to Future**

Think about one concrete step you can take in the coming weeks that connects your present self to the future work self you have described. This step might involve learning something, reaching out to someone, applying somewhere or changing a habit. Describe this step in detail. Why did you choose it? How does it bring you closer to your future work self?

**S5.2 Active Control Condition**

*Instructions were matched to the intervention condition on session duration, frequency, response format and expected word count. Topics were selected to be engaging and to require structured planning but to contain no occupational or career content.*

**Session 1: Planning a Journey**

Imagine you can take a trip to any destination you choose. Where would you go? How would you get there? What would you do each day? Describe this trip in as much detail as you can. Include practical details such as transportation and accommodation as well as the experiences you hope to have.

**Session 2: Planning a Shared Living Arrangement**

Imagine you are setting up a shared living arrangement with friends or classmates. What kind of space would you look for? How would you divide responsibilities? What rules or agreements would help everyone get along? Describe this arrangement in detail.

**Session 3: Planning a Personal Learning Project**

Think of a skill or topic you have always wanted to learn that is unrelated to your studies. How would you go about learning it? What resources would you use? How would you organize your time? Describe your learning plan in detail.

**Session 4: Planning a Community Activity**

Imagine you are organizing a community activity or event for your neighbourhood or campus. What would this activity involve? Who would participate? How would you arrange the logistics? Describe the planning process and the event itself in detail.

**Supplementary Table S6. Recruitment and Allocation by Participating University**

This table is new in the present revision and is added in response to the request of Reviewer 1 for the number of participants recruited from each university. Randomisation was stratified by university and by degree level, so allocation is closely balanced across the three arms within every site and the treatment contrasts are not confounded with university-level differences. Institutional tier was additionally entered as a covariate in every model. Clustering was not modelled with a random intercept, for the reasons given in Section 2.7 of the main manuscript.

| **University** | **Tier** | **Screened** | **Randomised** | **FWS writing** | **Active control** | **Waitlist** |
| --- | --- | --- | --- | --- | --- | --- |
| University 1 | Key (211 / Double First Class) | 178 | 141 | 48 | 46 | 47 |
| University 2 | Key (211 / Double First Class) | 152 | 102 | 34 | 34 | 34 |
| University 3 | Regional | 156 | 132 | 44 | 44 | 44 |
| University 4 | Regional | 126 | 111 | 36 | 38 | 37 |
| Total |  | 612 | 486 | 162 | 162 | 162 |

*Note. Institution names are given as University 1 to University 4 rather than by name, consistent with the ethics approval for the trial. Permuted blocks of size six were used within strata defined by university and degree level; because the final block within a stratum may be incomplete, the three arms are closely but not exactly balanced within each site. The key-university totals by arm (82, 80 and 81) correspond to the institutional tier counts reported in Table 4 of the main manuscript.*

**Supplementary Table S7. Attrition Analysis**

This table is new in the present revision and is added in response to the request of Reviewer 1 for a clearer missing-data analysis. Full information maximum likelihood yields consistent estimates when data are missing at random, which is a weaker condition than missing completely at random and which is not testable against the observed data (Graham, 2009). Panel A reports observed retention by arm and wave. Panel B reports the tests bearing on the plausibility of the missing at random assumption. Data missing not at random cannot be excluded by any of these procedures, and this is recorded as the twelfth limitation of the trial.

**Panel A. Retention and losses by arm and wave**

| **Wave** | **FWS writing** | **Active control** | **Waitlist** | **Total retained** | **Retention %** |
| --- | --- | --- | --- | --- | --- |
| Randomised (T1) | 162 | 162 | 162 | 486 | 100.0 |
| Post-intervention (T2) | 154 (lost 8) | 151 (lost 11) | 152 (lost 10) | 457 | 94.0 |
| 1-month follow-up (T3) | 147 (lost 7) | 143 (lost 8) | 146 (lost 6) | 436 | 89.7 |
| 3-month follow-up (T4) | 141 (lost 6) | 135 (lost 8) | 139 (lost 7) | 415 | 85.4 |

**Panel B. Tests bearing on the missing-data mechanism**

| **Test** | **Result** | **Interpretation** |
| --- | --- | --- |
| Little's MCAR test (all waves; Little, 1988) | chi-square = 18.72, df = 14, p = .176 | Reported for completeness. A non-significant result does not establish that data are missing completely at random. |
| Logistic regression of dropout on condition, all baseline demographic characteristics and the baseline value of every outcome, estimated separately at T2, T3 and T4 | No predictor significant at any wave; all p > .22 | No observed baseline variable is associated with dropout. Variables associated with missingness were retained in the analysis models. |
| Test of differential attrition across the three arms, adjusted for the covariates above, at the final wave | chi-square(2) = 1.04, p = .59 | Attrition does not differ across conditions. |

*Note. The figure in parentheses is the number lost since the preceding wave rather than a cumulative total. Retention percentages are computed against the 486 randomised participants. Because full information maximum likelihood was used, all 486 randomised participants contribute to every intention-to-treat model and the number analysed in each arm is 162 at every wave; the counts above are available-case numbers.*

**Supplementary references**

Brislin, R. W. (1970). Back-translation for cross-cultural research. Journal of Cross-Cultural Psychology, 1, 185-216.

Graham, J. W. (2009). Missing data analysis: Making it work in the real world. Annual Review of Psychology, 60, 549-576.

Little, R. J. A. (1988). A test of missing completely at random for multivariate data with missing values. Journal of the American Statistical Association, 83, 1198-1202.

Luyckx, K., Schwartz, S. J., Berzonsky, M. D., Soenens, B., Vansteenkiste, M., Smits, I., et al. (2008). Capturing ruminative exploration: Extending the four-dimensional model of identity formation in late adolescence. Journal of Research in Personality, 42, 58-82.
